# The Phylogenetic Relationship of Lamiinae (Coleoptera: Cerambycidae) Using Mitochondrial Genomes

**DOI:** 10.3390/genes15010013

**Published:** 2023-12-20

**Authors:** Ke Li, Sheng-Wu Yu, Hao Hu, Yu-Feng Feng, Kenneth B. Storey, Yue Ma, Jia-Yong Zhang, Dan-Na Yu

**Affiliations:** 1 College of Life Science, Zhejiang Normal University, Jinhua 321004, China; 2 Longquan Protection Center of Qianjiangyuan-Baishanzu National Park, Lishui 323700, China; 3 Department of Biology, Carleton University, Ottawa, ON K1S 5B6, Canada; 4 Key Lab of Wildlife Biotechnology, Conservation and Utilization of Zhejiang Province, Zhejiang Normal University, Jinhua 321004, China

**Keywords:** Lamiinae, intergenic spacer, phylogeny, mitochondrial genomes

## Abstract

**Simple Summary:**

Lamiinae is the largest subfamily among the Cerambycidae (longhorn beetles), and its members are distributed worldwide. The monophyly of Lamiinae is generally recognized, but there are still diverse ideas as to whether the tribes belonging to Lamiinae are monophylic. Ambiguous classification boundaries and the existence of synonyms are major issues leading to controversies over Lamiinae classification. It is not enough to conduct research solely on the morphological characteristics and simple molecular loci of longhorn beetles. Mitochondrial genomes have proven to be reliable markers and can shed more light on phylogenetic relationships among Lamiinae. The present study resolved infra-subfamilial relationships among Lamiinae and provides more mitochondrial data for further phylogenetic research on longhorn beetles.

**Abstract:**

Lamiinae is the largest subfamily of the Cerambycidae (longhorn beetles), with approximately 21,863 described species. Previous phylogenetic studies of Lamiinae showed that this subfamily was monophyletic, but the relationship between the tribes of Lamiinae is still controversial. Partial molecular data and species morphological characteristics are not sufficient to resolve species phylogenetic studies perfectly. At the same time, the full mitochondrial genome contains more comprehensive genetic data. Benefiting from the development of next-generation sequencing (NGS), mitochondrial genomes can be easily acquired and used as reliable molecular markers to investigate phylogenetic relationships within Cerambycidae. Using NGS technology, we obtained 11 mitochondrial genome sequences of Lamiinae species. Based on this newly generated mitochondrial genome dataset matrix, we reconstructed the phylogeny of Lamiinae. The Bayesian Inference and Maximum Likelihood analyses strongly support the monophyly of four tribes (Lamiini, Batocerini, Mesosini, and Saperdini), whereas the tribe Acanthocinini was identified as paraphyletic. Other mitochondrial structural features were also observed: the start codon in the nad1 gene of all 11 mitochondrial genomes is TTG; 17–22 bp intergenic spacers (IGS) with a ‘TACTA’ motif were found between *trnS2* and nad1. Moreover, two long IGS were found in *Mesosa myops* and *Batocera* sp. Tandem repeats were found in the IGS of *Batocera* sp.

## 1. Introduction

As the largest and most widespread insect group, the taxonomy and phylogeny of Coleoptera have been extensively studied. Normally, Coleoptera is divided into four suborders: Archostemata, Adephaga, Myxophaga, and Polyphaga [1]. There are 38,874 described species of Cerambycidae (Coleoptera: Polyphaga) to date, according to the “Titan database about Cerambycidae” (http://titan.gbif.fr/sel_sous_famille.php, accessed on 31 January 2023) [2]. It has been reported that such massive species diversity is linked to the development of angiosperms [3]. The most primitive Cerambycidae may have appeared on Earth in the Late Jurassic period [4], but the prosperity of Cerambycidae is estimated to have occurred between 150 and 64 million years ago [5]. Because there are so many species of longhorn beetles, the number of subfamilies of longhorn beetles is still controversial. Lawrence proposed that there were 13 subfamilies [6], whereas Bouchard et al. classified Cerambycidae into nine subfamilies [7]. Švácha and Lawrence revised the Cerambycidae as eight subfamilies [8]. Overall, Cerambycinae, Dorcasominae, Lamiinae, Lepturinae, Parandrinae, Prioninae, and Spondylidinae are recognized by most researchers [6,7,8,9,10,11] (Table 1).

With the exception of Antarctica, Lamiinae are found in all biogeographic regions, from low to high latitudes [12]. The variety of adult longhorn beetle morphologies is extensive. For example, the body length of *Cyrtinus pygmaeus* (Lamiinae: Cyrtinini) is just 2 mm, but the body length of *Acrocinus longimanus* (Lamiinae: Acrocinini) can reach 80 mm. Lamiinae species feed on specific plant tissues, such as leaves, branches, and shells, and a few feed on flowers [13]. The Lamiinae variety is extraordinarily diverse, with 21,863 species in 86 tribes (http://titan.gbif.fr/index.html, accessed on 31 January 2023). There are 28 tribes, 307 genera, and approximately 1636 species (including subspecies) in China [14,15,16]. The problem of Lamiinae is mainly focused on the monophyly of the tribal level. Souza et al. conducted a thorough developmental evaluation of the tribes of Lamiinae for the first time under a molecular phylogenetic framework [17]. Their study supported the monophyly of Lamiinae and the monophyly of the 11 tribes within this subfamily. But the boundaries of 15 tribes within Lamiinae require to be revised: Acanthocinini, Acanthoderini, Agapanthiini, Apomecynini, Desmiphorini, Dorcaschematini, Enicodini, Hemilophini, Monochamini, Onciderini, Parmenini, Phytoeciini, Pogonocherini, Pteropliini, and Saperdini [17]. Laurene et al. subsequently followed with comprehensive phylogenetic investigations of the Lamiinae tribes in Australia [18], and they also concerned the monophyly of Lamiinae while negating the monophyly of Lamiinae at the tribal level. This provided new clues to the origin in time and the relationship of Lamiinae in Australia. In the research of Napp [19], Raje et al. [20], and Haddad et al. [21], morphological data, mitochondrial genome data, and nuclear gene data were utilized for assessing the phylogenetic relationships within Lamiinae, that confirmed the monophyly of Lamiinae.

Next-generation sequencing (NGS) technologies have reduced the time, costs, and complexity of obtaining whole mitochondrial genome data [22]. As eukaryotic organelles, mitochondria have a double-stranded circular DNA structure. The mitochondrial genome is very small, with only 37 genes [23]. It is characterized by maternal inheritance, relatively conserved genes, and a high evolutionary rate, this latter feature serving as a valuable molecular marker for studying phylogenetic relationships [24,25]. Ayivi et al. sorted out the phylogenetic relationships of subfamilies within Scarabaeidae using the 18 sequenced mitochondrial genomes of scarabaei beetles [26]. Yu et al. supported the Chiastomyaria hypothesis using the mitochondrial genomes based on three different outgroups [27]. Zhang et al. figured out part of the phylogeny and taxonomy in Culicomorpha using PCGs, tRNA, and rRNA genes of mitochondrial genomes [28]. Additionally, mitochondrial genomes have also been used as molecular markers in the phylogeny of Mantodea [29,30], Phasmatodea [31,32], Archaeognatha [33], Ephemeroptera [34], etc., with remarkable results.

To date, eighty complete or partial mitochondrial genomes of Lamiinae (≥13,854 bp) have already been published in the NCBI [10,11,35,36,37,38,39,40,41,42,43,44,45,46,47,48,49,50]. The position of the Lamiinae subfamily has been clearly delineated, but the relationships of Lamiinae tribes remain vague [17,51]. For our present study, we acquired mitochondrial genome sequences for 147 Coleopteran species from the NCBI and combined these with the genome data for the 11 Lamiinae species provided in the current study. The present study investigates (1) the gene structure of the 11 mitochondrial genomes reported in this study, (2) the monophyly of subfamilies of Cerambycidae, and (3) the monophyly of tribes of Lamiinae.

## 2. Materials and Methods

### 2.1. Taxon Sampling and Mitochondrial Genome Sequencing

Samples for this study were collected in Jilin city, Jilin province and Wenshan county, Yunnan province, China (Table 2). The latitude and longitude of the two sample sites are Jilin city (E 126.57°, N 43.87°) and Wenshan County (E 104.24°, N 23.40°). The samples are preserved in the Museum of Zoology, Zhejiang Normal University, China. Tissue extraction from all samples was performed immediately, and the remaining insect tissue was stored at −20 °C in 100% ethanol. External morphological characteristics of samples were checked by Dr. JY Zhang and confirmed by DNA barcoding for the cox1 gene in NCBI and BOLD system v4 (Barcode of Life Data System, http://www.boldsystems.org/, accessed on 15 April 2023). Total DNA was extracted from thorax muscle using Ezup Pillar Animal Genome DNA Purification Reactor Box (Biotech, Shanghai, China). Whole genome DNA from all samples was delivered to BGI Technology Corporation Shenzhen, China) for NGS sequencing on the Illumina MiSeq platform using the shotgun method. FastQC was used for the quality control process. The mitochondrial genome was assembled using GetOrganelle based on paired-end clean reads {maximum extension rounds (suggested > 2), default = 10; SPAdes kmer setting, default:21,45,65,85,105} [22,52,53]. *Paraglenea fortunei* (MW858148), *Nortia carinicollis* (MK863508), *Monochamus alternatus* (MT547196), and *Peithona prionoides* (MN473095) were selected as reference sequences to aid the sequence assemblage.

### 2.2. Mitochondrial Genome Annotation and Sequence Analyses

We employed the Mitos2 web server to provide annotation of the mitochondrial genomes (http://mitos.bioinf.uni-leipzig.de/index.py, accessed on 15 April 2023) [54,55]. The position and secondary structure of the tRNAs were validated by tRNAScan-SE (http://lowelab.ucsc.edu/tRNAscan-SE/index.htm, accessed on 20 April 2023) [56]. To determine the 12S and 16S RNAs, we compared the homologous genes of other Lamiinae species using Mega 7.0 [57]. According to the invertebrate genetic code, 13 PCGs (protein-coding genes) from all species were identified as open reading frames using Mega 7.0. PCGs, which lack canonical start codons and stop codons in translation, were adjusted by MEGA 7.0. Finally, serial repeat sequences in the control region were predicted by an online network server software: Serial Repeat Sequence Finder Version 4.09 (https://tandem.bu.edu/trf/trf.basic.submit.html, accessed on 20 May 2023) [58]. After using PrimerPremier 5 to design sequence-specific primers, the long intergenic spacer (IGS) was amplified and sequenced using specific primers and Sanger sequencing to check whether the repeat regions existed [59,60].

We used the CG View online server to draw a circular graph of 11 samples (http://cgview.ca/, accessed on 25 March 2023) [61,62]. Analysis of nucleotide composition skew used the formula: AT-skew = (A − T)/(A + T) and GC-skew = (G − C)/(G + C) [63]. Codon usage and relative synonymous codon usage (RSCU) were analyzed by PhyloSuite2 and graphed in Adobe Illustrator [64,65].

### 2.3. Phylogenetic Analyses

Phylogenetic analyses were performed on 155 species of Cerambycidae and 3 species of Cucujidae beetles, with the latter used as outgroups. Among the 155 mitochondrial genomes of Cerambycidae, 144 mitochondrial genomes were downloaded from the NCBI (Table S1), and the other 11 species were sequenced in this study. Nucleotides of all 13 protein-coding DNA sequences were extracted using Phylosuite, removing stop codons and spacers [64]. Then, the codon saturation of the sequence was tested by DAMBE (<0.8) [66]. The best model was selected for Bayesian Inference and Maximum Likelihood using Partitionfinder version 2.2.1, and best-fit substitution models for the nucleotide datasets are shown in Table S2 [67]. For Bayesian Inference analysis in MrBayes, the best-fit partition models for the nucleotide datasets were GTR + I + G. MrBayes and IQ-tree were utilized for Bayesian Inference and Maximum Likelihood, respectively, to generate phylogenetic trees [68,69]. MrBayes was used for phylogenetic tree construction, with default parameters and 5 × 10^6^ Markov Chain Monte Carlo (MCMC) generations with sampling every 1000 generations. The first 25% of sample data were deleted as burn-in, and convergence was defined as an average standard deviation of split frequencies less than 0.01. The best-fit partition model for Maximum Likelihood using IQ-tree was also GTR + I + G for the nucleotide datasets, too. Maximum Likelihood phylogenetic analysis was performed, with branch support for each node evaluated under 1000 rapid replications.

## 3. Results

### 3.1. General Characteristics of 11 New Mitochondrial Genomes

In this study, 11 Lamiinae mitochondrial genomes were sequenced, and the mitochondrial genome structures are depicted in Figure S1. Except for two partial mitochondrial genomes of *Rondibilis* sp. and *Macrochenus isabellinus*, the remaining nine mitochondrial genomes were all complete. The profile compositions of all samples are identical, with a typical Coleoptera mitochondrial genome arrangement (e.g., with the trnW-trnC-trnY cluster). One control region (CR), two ribosomal RNA genes (*12S* and *16S RNA*), 22 transfer RNAs (tRNAs), and 13 protein-coding genes (PCGs) were present in all mitogenomes of this study (Table S3). The nucleotides found in Lamiinae genomes were biased towards A and T, the two nucleotides accounting for 74.6–79.1% of the base pairs. The G-C skew was negative for the entire mitochondrial genome (Table S4). The A-T and G-C skew values were both negative in the H-strand of PCGs (Table S5). The number of overlapping sections in the mitochondrial genomes ranged from 10 to 14, with the overlapping sizes varying from 1 to 8 bp. All mitogenomes from the 11 species contained 13 PGCs (11,058 bp) and accounted for 59.78% to 71.48% of the mitogenome. In all of the sequences, the H-strand encoded nine PCGs (atp6, atp8, cox1, cox2, cox3, cytb, nad2, nad3, and nad6), whereas the L-strand encoded the remaining four PCGs (nad1, nad4, nad4l, and nad5). The start codon was AAN in most cox1 genes (except for *Mesosa myops*, which used ATT) (Table S6). The start codon of nad1 was TTG. All other PGCs used ATN as the start codon. There are four types of stop codons: T, TA, TAG, and TAA. The relative synonymous codon usage of the 11 Lamiinae mitochondrial genomes is shown in Figure S2. Except for stop codons, the total number of codons ranged from 3696 to 3704. Leu2 (UUA), Ile (AUU), Phe (UUU), and Met (AUA) were the most regularly used codons. All codons had either A or T nucleotides, indicating that a high AT mutation bias had a discernible effect on codon usage.

Two ribosomal RNAs (*16S* and *12S RNA*) were identified in all mitogenomes (Table S3). Among the 11 mitochondrial genomes, *16S RNA* sizes ranged from 1270 bp for *Rondibilis* sp. to 1284 bp for *Batocera* sp., and *12S RNA* sizes ranged from 776 bp for *Pharsalia subgemmata* to 845 bp for *Acalolepta permutans*. Except for *trnS1*, the secondary structure of the tRNA was the classical clover leaf type. The *trnS1* of all 11 sequences lacked the dihydrouridine (DHU) arm, resulting in a simple loop at this location (Figures S3–S13). Mismatched pairs, such as G-U and U-G, occurred in the tRNA stems of 11 species, all of which were found in four different stems.

### 3.2. A+T-Rich Region

The length of the A+T-rich region ranged from 736 (*Batocera* sp.) to 3560 bp (*Me. myops*), and the A-T content ranged from 78% to 90% (Table S7). Tandem repeats were found in the A+T-rich region of 11 mitochondrial genomes (Tables S8–S18). The copy number of repeats in the A+T-rich region varied from 2 to 101 times, and the length of the repeat fragment of the A+T-rich region ranged from 1 to 395 bp (Tables S8–S18). Two types of poly-base stretches, TTTTTTTTT and AAAAAAAA, existed in the A+T-rich region (Table S7). A poly-T stretch was found in 11 A+T-rich regions with lengths ranging from 9 to 33 bp. In addition, a 16-bp poly-A stretch was discovered in the A+T-rich region of *Ma. isabellinus*, as well as two 9-bp poly-A stretches in the A+T-rich region of *P. subgemmata*.

### 3.3. Intergenic Spacers

**Figure 1:**
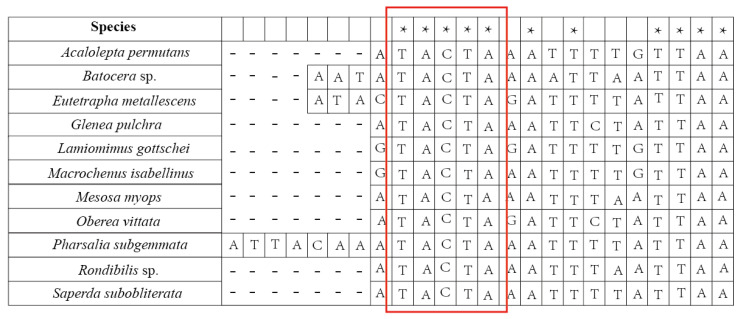
Alignment of the intergenic spacers between *trnS2* and nad1, with a 5-bp consensus motif (red box).

**Figure 2:**
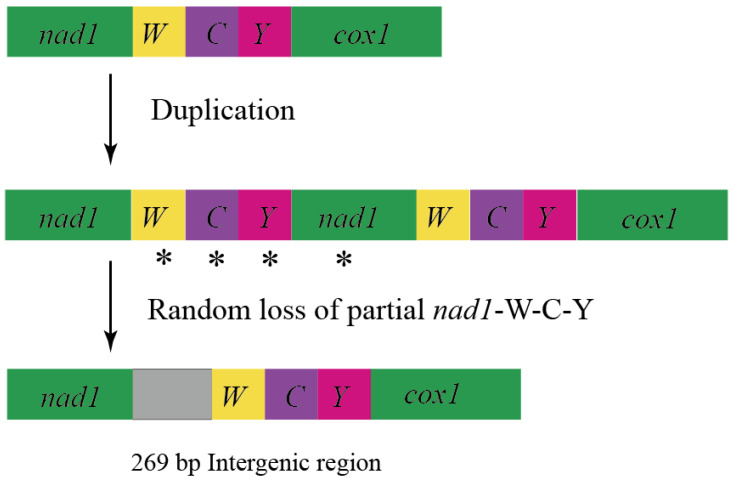
Putative mechanism of long IGS in the mitochondrial genome of *Mesosa myops* under the duplication/random loss model. The random losses of partial genes are marked with *.

**Figure 3:**
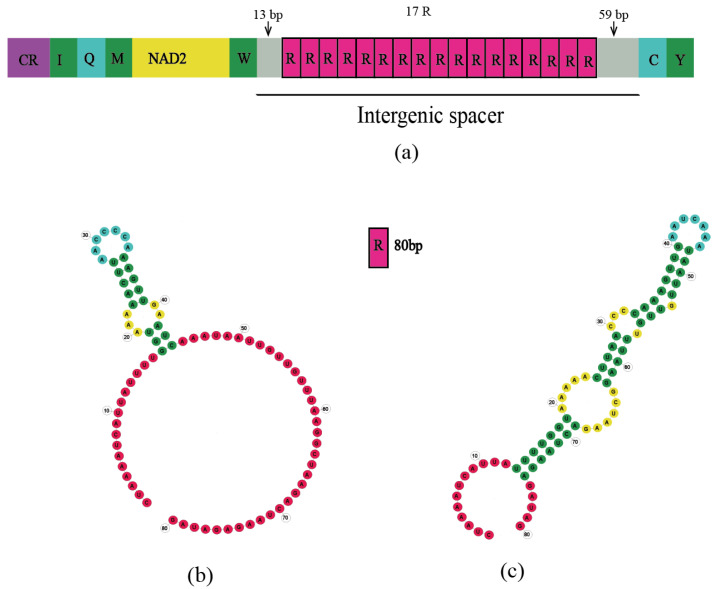
(**a**) Schematic of long IGS of *Batocera* sp., with the IGS located between *trnW* and *trnC*. R (80 bp) represents the repeat segments, with a total of 17 repeat segments. (**b**,**c**) are two types of stem-loop structures of 80-bp R.

Intergenic spacers (IGSs) were found in the coding regions of all 11 sequences. Short IGS regions included a 7-bp IGS located between trnI and trnQ in *Rondibilis* sp. and a 10-bp IGS located in *Lamiomimus gottschei*. The IGSs between *trnS2* and nad1 were 20 bp in *Eutetrapha metallescens* and *Batocera* sp., 24 bp in *P. subgemmata* and 17 bp in the remaining sequences. A 5-bp consensus motif (TACTA) existed in the IGS of 11 species between *trnS2* and nad1 (Figure 1). Among long IGSs, the most notable were the 269-bp IGS between nad2 and *trnW* in *Me. myops* (Figure 2, Figures S14 and S15), and the 1428-bp IGS between *trnW* and *trnC* in *Batocera* sp. (Figure 3, Tables S19 and S20). The long IGS in *Me. myops* contained 82% of the A-T content without any repetitive sequence, whereas the IGS in *Batocera* sp. contained 72% of the A-T content with repetitive sequences. The IGS of *Batocera* sp. had 17 repeats with two types of stem-loop structures. We verified the existence of these two long IGSs by Sanger sequencing using the specific primers (Table S21).

### 3.4. Phylogenetic Analyses

**Figure 4:**
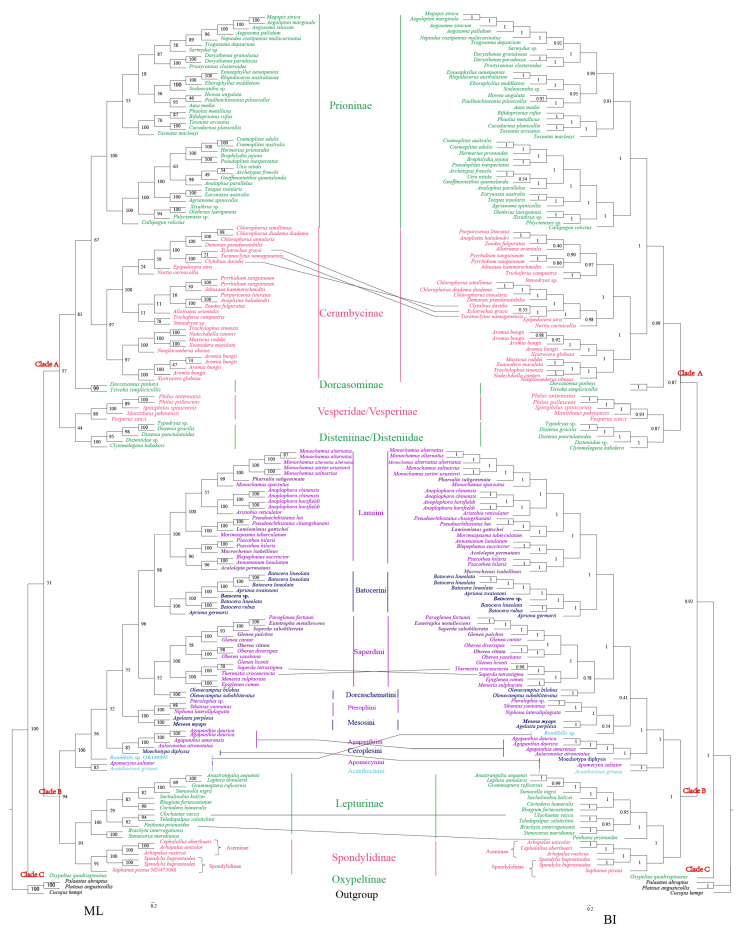
Phylogenetic relationships inferred by the Maximum Likelihood (**Left**) and Bayesian Inference (**Right**) methods based on nucleotide datasets. Numbers on nodes are the posterior probabilities of Bayesian Inference and bootstrap values of Maximum Likelihood. Different branch colors correspond to subfamily names (purple, dark blue, and light blue represent Lamiinae, and the text represents tribe names; green and pink are non-Lamiinae, and the text represents the subfamily names). The red text represents the branch name (Clade A, Clade B, Clade C).

Bayesian Inference and Maximum Likelihood were conducted for all concatenated data matrices. The topologies derived from Bayesian Inference and Maximum Likelihood were similar (Figure 4). The nine subfamilies of Cerambycidae were divided into three clades. Clade A showed a topology of (((Prioninae + Cerambycinae) + Dorcasominae) + (Disteniinae + Vesperinae)), with Prioninae and Cerambycinae being sister groups. These two subfamilies were clustered into one clade with Dorcasominae. Disteniinae and Vesperinae are sister groups, clustered in one clade with ((Prioninae + Cerambycinae) + Dorcasominae). Clade B showed a topology of (Lamiinae + (Lepturinae + Spondylidinae)) where Lepturinae and Spondylidinae are sister groups and then clustered in one clade with Lamiinae. Newly sequenced mitochondrial genomes in this study were situated in Clade B. Clade C with a topology of Oxypeltinae was in a near basal position and was sister to Clade A + Clade B. Nine subfamilies appear to be monophyletic.

In the Bayesian Inference (BI) and Maximum Likelihood (ML) results, there are four different clustering relationships. (1) The clustering relationships of *Clytobius davidis*, *Xylotrechus grayii*, and *Turanoclytus namaganensis* are (*C*. *davidis*+ (*X*. *grayii + T*. *namaganensis*)) in the ML tree, whereas the relationship in the BI tree was ((*C*. *davidis + X*. *grayii*) *+ T*. *namaganensis*). (2) The clustering relationships of *Glenea licenti*, *Saperda tetrastigma*, and *Thermistis croceocincta* are ((*G. licenti* + *S. tetrastigma*) + *T. croceocincta*) in ML tree, whereas ((*G. licenti* + *T. croceocincta*) + *S. tetrastigma*) in BI tree. (3) The positions of *Rondibilis* sp. in the BI and ML trees are different. *Rondibilis* sp. is a separate branch in the ML tree, whereas *Rondibilis* sp. and (Pteropliini + Mesosini) are clustered into one clade in the BI tree. (4) *Peithona prionoide* and (*Teledapalpus zolotichini*+ *Ulochaetes vacca*) are clustered in one clade in the ML tree, whereas *Peithona prionoides* is in the outermost layer of the Lepturinae branch in the BI tree.

All new species sequences obtained in this study belonged to five tribes of Lamiinae: (1) *A. permutans, L. gottschei*, *Ma. isabellinus*, *P. subgemmata*, (Lamiini); (2) *Batocera* sp. (Batocerini); (3) *E. metallescens*, *G. pulchra*, *O. vittata*, *S. subobliterata* (Saperdini); (4) *Me. myops* (Mesosini); and (5) *Rondibilis* sp. (Acanthocinini). In this study, Lamiinae totally contained nine tribes. The ML and BI trees show that the eight tribes are monophyletic (Figure 4): Lamiini, Batocerini, Saperdini, Dorcaschematini, Pteropliini, Mesosini, Agapanthiini, and Ceroplesini. However, Acanthocinini is Paraphyletic (Figure 4). Apomecynini has only one sequence and is clustered in one clade with Acanthocinini. Therefore, we cannot clarify the monophyly of Apomecynini.

## 4. Discussion

### 4.1. General Features of Mitochondrial Genomes

Each mitochondrial genome in this study has a similar compositional profile, typical gene arrangement, and orientation that are shared by most coleopteran insects [70,71]. Except for *Me. myops*, the lengths of the remaining 10 mitochondrial genomes are within the sequence length range that NCBI has published (Table S4).

The order of genes in Lamiinae was the same as the ancestral orientation order (trnI-trnQ-trnM) [23]. The start codon for most protein-coding genes was ATN, whereas the start codon for nad1 was TTG, and we assume that cox1 uses AAC as the start codon (except for *Me. myops*, where cox1 starts with ATT) [42,72]. The aberrant tRNA (*trnS1*) has been shown to be functional, but it is slightly less effective than regular tRNAs. The *trnS1* in Cerambycidae uses TCT as an anticodon, and many other coleopteran mitochondrial genomes also use TCT as an anticodon [73,74]. Stop codons were generally TAA or TAG, but incomplete stop codons (TA or T) occurred in some species (Table S6). During mRNA maturation, posttranscription polyadenylation can lead to the generation of just “T” [75,76]. The secondary structure of 21 tRNAs showed the typical triple-leaf structure, but trnS1 is not typical because it lacks the dihydrouridine (DHU) arm. This abnormal tRNA has been shown to be functional but with a slightly lower capacity than conventional tRNAs [77,78,79]. There were two types of mismatched pairs (G-U, U-G) found in the four different stems of tRNAs. It has been established that mismatched pairs can be revised by the editing process and may also represent abnormal matches [80].

### 4.2. A+T-Rich Region

The A+T-rich region, also known as the non-coding region, is rich in adenine and thymine nucleotides [81]. The high AT content and tandem repeats in this region make it difficult to identify this fragment by common sequencing methods (primers, Sanger, or short-read NGS) [82,83]. The definition of the A+T-rich region necessitates a precise assessment of repeat length, length frequency, and nucleotide composition, and we sequenced the majority of A+T-rich regions by next-generation sequencing (NGS) methods (Table S7). This is the largest noncoding region of the mitochondrial genome and a major factor in influencing variations in the length of the entire mitochondrial genome. This region contains transcription and replication beginning sites as well as important regulatory components [70].

Repeat copy numbers in the A+T-rich region varied from 2 to 102 times (Tables S8–S18). The most common model used to explain the wide range of repetitive units in the A+T-rich region is the illegitimate elongation model of Buroker et al. [84]. The length of these repeat units in the A+T-rich region ranged from 1 to 395 bp in different species (Tables S8–S18). The change in length in one repetitive sequence has a large impact on the size of the interspecies A+T-rich region and the entire mitochondrial genome [81,85]. For instance, the A+T-rich region of *Me. myops* contains 12 different types of repeat fragments, the longest of which is 395 bp and repeats seven times (Table S14). This is also the longest mitochondrial genome that we have sequenced, even surpassing the length of mitochondrial genomes in published databases on longhorn beetles. Actually, the length of the A+T-rich region will not be influenced by the copy number of repeat units. *E. metallescens* has a 2-bp tandem repeat unit that duplicates 102 times, but this region is only 868 bp and the complete mitochondrial genome is 15,505 bp (Table S10). *Ma. isabellinus* showed a minimum number of tandem repeat units, but the A+T-rich region length is 1011 bp, and the whole length of the mitochondrial genome is 15,675 bp (Table S13). The length of *Ma. isabellinus* is bigger than *E. metallescens*. Therefore, we believe that the length of the repeat units in the A+T-rich region is the main factor affecting the length of the mitochondrial genome in Cerambycidae. The copy number of repeat units has a minor influence.

The A+T-rich region of the 11 sequences all had one or more poly-T stretches. “T-stretches” are a consecutive group of thymine nucleotides. We performed an alignment of poly-T and discovered a 9-bp consensus fragment located upstream of *trnI*. Previous research has shown that poly-T identifies the structural signals of proteins involved in the replication initiation process in holometabolic insects [86].

### 4.3. Intergenic Spacers

#### 4.3.1. Short IGS

Intergenic spacers, such as between *trnS2* and nad1, are well-known in Hymenoptera (Formicidae, Apoidea), Hemiptera (Triatominae), and Coleoptera (Lucanidae, Cleridae, Cerambycidae and Meloidae) [39,87,88,89,90,91,92,93]. In the present study, IGS was relatively short, with a length between 17 and 24 bp. In this region, there is a 5-bp consensus motif. Wang et al. found the same motif (TACTA) in the mitochondrial genome of Cerambycidae [39,93], and this motif is also conserved in Coleoptera [42,93]. At the same location, a 6 bp motif (THACWW) was found in Hymenoptera, as well as the motif (ATACTAA) in Lepidoptera. This suggests that this region is conserved and found in a majority of the insect mitochondrial genomes [94,95,96]. This motif is considered a possible recognition site for the mtTERM protein (transcriptional stop peptide) [97]. We infer the motif can be a molecular marker to verify the different kinds of insects.

#### 4.3.2. Long IGS of *Mesosa myops*

Long intergenic spacers (>50 bp) have been reported in various insects, but most IGS is located between *trnS* and nad1 [89,90,91,92]. However, long IGSs are found elsewhere in the mitochondrial genomes of some insect groups: Cerambycidae (Coleoptera), Meloidae (Coleoptera), Scirtidae (Coleoptera), Chaetosomatidae (Coleoptera), Priasilphidae (Coleoptera), Ectobiidae (Blattodea), and Formicidae (Hymenoptera) [39,89,96,98,99].

There are two generally accepted evolutionary mechanisms to explain the appearance of IGSs in the mitochondrial genome: (a) the duplication/random loss model and (b) lipped-strand mispairing. We could not discover homologous sequences at either terminal of the 269-bp IGS, so it was difficult to explain its formation by slip mispairing. Wang et al. found a 184 bp IGS in the longhorn beetle between *trnC* and *trnY*. They used a duplication/random loss model to explain its formation [39]. The IGS found by Du et al. in Meloidae is located between *trnW* and *trnC* and can be explained by the same model. The homology of IGS and the original tRNAs is quite low. Repeat/random loss events in *Hycleus* may occur comparatively early and delete many nucleotides during the random loss stage [96]. Yan et al. proposed that IGSs may originate from replication of the 3′ end of *12S RNA* when the DNA double helix unwinds. Subsequently, random loss of some duplicated genes occurred, leaving behind residues that ultimately constituted the IGS observed in *E. splendens* [98]. In this study, a 269-bp IGS was located between *trnW* and *trnC* in *Me. myops* without repetitive sequences. We compared this 269-bp IGS with 37 genes in *Me. myops* and found that their homology was very low. Combined with the studies of Wang et al. [39] and Du et al. [96], we also believe that the duplication/random loss model is the best to explain the generation of the 269-bp IGS in *Me. myops* (Figure 2).

Sheffield et al. discovered IGSs between nad2 and *trnW* (177 bp) in *Cyphon* (Coleoptera: Scirtidae), between *trnD* and atp8 (83 bp) in *Chaetosoma* (Coleoptera: Chaetosomatidae), and between *trnY* and cox1 (203 bp) in *Priasilpha* (Coleoptera: Priasilphidae) [99]. Like these, the long IGS of *Me. myops* did not have tandem repeats and did not produce any significant BLAST results. The long IGS cannot translate into any amino acid sequence without a stop codon. It can form two types of stem-loop structures (Figure S14). But its function needs further study [99]. Until now, there have been few studies of the function of IGS in Coleoptera, so we turned our attention to other insects. Rodovalho et al. demonstrated that IGSs were highly variable and informative for subspecies-level studies, and it might help to distinguish the sibling species of Attini ants [89]. Therefore, determining the positions of IGS in the mitochondrial genome of the Attini ant might be useful for phylogenetic analyses [82]. The size of the IGS between cox1 and cox2 increased sequentially in Attini ants, honey ants, and bees, implying that the change in IGS size can probably be used as an evolutionary marker for social insects [87,100,101,102]. These IGSs of unknown function may be valuable in describing genome evolution and distinguishing closely related species or individuals. However, the small number of Cerambycidae samples with IGS makes it hard to fully confirm whether IGS in the mitochondrial genome of Cerambycidae can be used as markers for phylogenetic analysis. Meanwhile, there is no gene rearrangement found in Cerambycidae, so we believe that IGS has little effect on gene rearrangement in Cerambycidae. We favor the 269-bp IGS in *Me. myops* as another factor affecting the length of the entire sequence in addition to the A+T-rich region [39,89] (Figure S15).

#### 4.3.3. Long IGS of *Batocera* sp.

We discovered a very long IGS in *Batocera* sp. (Figure 3). However, the examination of mitochondrial genomes of other members of the genus *Batocera* did not find similar structures: *B. lineolata* (MW629558, MZ073344, MF521888) and *B. rubus* (OM161963). Similar sequences are also not present on the NCBI. The long IGS of *Batocera* sp., a 1428 bp spacer with 17 repeat fragments and high AT content, is between *trnW* and *trnC* (note: these are usually characteristic of the A+T-rich region) (Figure 3, Table S19). Similar structures have been found in *Pyrocoelia rufa* [74], Evania appendigaster [94], and Metopodontus blanchardi [103,104] (Table S20).

In the studies of Bae et al. [74], Wei et al. [94], and Kim et al. [104], the authors did not offer an explanation for how this IGS was formed or what its function was. Xu et al. also performed an analysis of large IGS in the genus Prosopocoilus (Lucanidae) [103] and discovered IGSs between *trnI* and *trnQ* in the mitochondrial genome of genus *Prosopocoilus* (Lucanidae), with varying lengths from 375 bp to 4051 bp [103]. Such IGSs have only been found in the mitochondrial genomes of this genus. Hence, this feature may be synapomorphic for members of the genus *Prosopocoilus*, supporting the taxonomic positions of the other 195 existing species within this genus, as Xu et al. suggested. Hence, this IGS may be a practical molecular marker to distinguish *Prosopocoilus* from its closely related and indistinguishable genera. However, a 1428-bp IGS in *Batocera* sp. is a special case. We did not find a similar structure between trnW and trnC in other species of the genus *Batocera* and no similar structure has been found in other longhorn beetles. Therefore, the 1428-bp IGS cannot work as a taxonomic marker to distinguish Cerambycidae species with similar appearance. We were also unable to determine the function and generation mechanism of this IGS. Ultimately, we tentatively identified IGS of *Batocera* sp. as a special noncoding region for a specific species. We await the results of future studies to determine its function and production mechanism.

### 4.4. Phylogenetic Analyses

#### 4.4.1. Phylogenetic Analyses of Subfamily

We used a total of 158 longhorn beetle mitochondrial genomes for phylogenetic analysis, representing 11 subfamilies (Figure 4): Lamiinae, Lepturinae, Necydalinae, Aseminae, Spondylidinae, Prioninae, Cerambycinae, Dorcasominae, Disteniinae (=Disteniidae) [105], Vesperinae (=Vesperidae) [11], and Oxypeltinae (=Oxypeltidae) [21]. The phylogenetic tree shows that longhorn beetles are divided into three main groups: Clade A, Clade B, and Clade C. This study generalizes Necydalinae to Lepturinae and Aseminae to Spondylidinae and supports the monophyly of nine subfamilies of Cerambycidae and the family-level status of Oxypeltidae.

The monophyly of Prioninae is highly recognized in clade A, but the monophyly of Cerambycinae in the clade is controversial [5,11,17,39,106]. Previous classifications of Cerambycinae by adult or larval morphologies resulted in its being misassigned into other subfamilies and, thus, did not restore monophyly with this subfamily [8]. However, Cerambycinae classification has become clearer as the amount of molecular data increases. Many researchers have utilized various species and numbers of molecular loci to recover the monophyly of Cerambycinae [4,21,107,108,109,110]. Dorcasominae and (Prioninae + Cerambycinae) are sister groups in this clade, and Dorcasominae is recognized as monophyletic [7]. However, Disteniidae and Vesperidae are supported as family ranks [11,15,111,112,113,114,115,116,117,118,119]. Following Crowson and Švácha and Danilevsky, the current study supports the monophyly of Vesperidae, which is the sister group of Disteniidae [114,118]. Haddad et al. restored Disteniidae as a sister to Cerambycidae *s. s.* (sensu stricto) and Vesperidae as a sister to this clade [21]. In this study, we supported the monophyly of subfamilies Prioninae, Cerambycinae, Dorcasominae, Vesperidae, and Disteniidae. We cannot infer the family rank of Vesperidae and Disteniidae.

In Clade B, the monophyly of Lamiinae is strongly supported (BP = 99, PP = 0.99), which is consistent with the results of many previous studies [11,17,18,39]. Necydalinae nested within Lepturinae, and it clustered with *Teledapalpus zolotichini* into one clade. In the study of Souza et al., Necydalinae was recovered as a monophylum species nested in Lepturinae *s. s.* (sensu stricto). The authors considered Necydalinae to be a tribe of Lepturinae (tribe Necydalini) [120,121,122,123,124,125]. Aseminae gathered together with Spondylidinae and were considered independent subfamilies in previous studies [19,111,112,113,126], but this division is not supported from the perspective of larval morphology and molecular studies (mitochondrial 16S rDNA) [20,114,127,128]. Recent studies have abandoned the subfamily rank of Aseminae, assigned Aseminae to the subfamily Spondylidinae, and supported the monophyly of Spondylidinae [5,11,21,129,130,131].

In clade C, Oxypeltidae is a reciprocal sister group with (clade A + clade B). Many studies in recent years support that Cerambycidae *s. l.* (sensu lato) consists of Cerambycidae *s. s.* (sensu stricto), Disteniidae, Oxypeltidae, and Vesperidae. However, Oxypeltidae has been considered to be a family-level in some studies [11,18,21,39]. We also support the family rank of Oxypeltidae in this study.

#### 4.4.2. Tribal Classification in Lamiinae

The data presented in the current study indicate the monophyly of the Lamiinae while casting doubt on the monophyly of the Lamiinae tribe. The analyses of Bayesian Inference and Maximum Likelihood support the conclusion that seven of the ten tribes appear to be monophyletic: Lamiini, Batocerini, Dorcaschematini, Pteropliini, Mesosini, Agapanthiini, and Ceroplesini. The Saperdini branch shows subtle differences in the clustering relationships of Bayesian Inference and Maximum Likelihood: ((*Glenea licenti* + *Saperda tetrastigma*) + *Thermistis croceocincta*) (ML); (*Glenea licenti* + *Thermistis croceocincta*) + *Saperda tetrastigma*) (BI).

In the clade Lamiini, the Monochamini, Phrissomini, and Agniini are considered synonyms of Lamiini. Sumana et al. determined that the species *Ma. isabellinus* (Agniini) belonged to Lamiini [132], and Toki and Kubota also classified *Psacothea hilaris* (Agniini) as Lamiini [133]. Breuning first generalized the genus *Pseudoechthistatus* to Phrissomini, and some scholars agreed with this placement. Sama made Phrissomini a synonym for Lamiini [121,134]. Löbl and Smetana considered the genus *Pseudoechthistatus* (Phrissomini) to belong to Monochamini, and this tribe was separated from Lamiini [116]. *Pseudoechthistatus* was put into Lamiini by Bi and Lin, supporting the conclusion that Lamiini contains Monochamini [135]. In short, Agniini, Monochamini, and Phrissomini are synonymous with Lamiini [17,38,115]. Our findings similarly support this conclusion and the fact that Lamiini is monophyletic.

Souza et al. [17] first conducted a relatively intensive phylogenetic evaluation of Lamiinae, followed by Ashman et al. [18] and Shi et al. [10], who subsequently published two analyses of Lamiiniae. In the Lamiiniae, the monophyly of Batocerini, Mesosini, and Ceroplesini is definite, but controversies remain. Souza et al. [17] considered Batocerini, Mesosini, Lamiini, and Ceroplesini to be monophyletic, whereas Acanthocinini, Agapanthiini, Apomecynini, Dorcaschematini, Saperdini, and Pteropliini were paraphyletic. Acanthocinini and Apomecynini are widely distributed in the topology. Ashman et al. [18] believed that the Saperdini are monophyletic, whereas the Acanthocinini, Apomecynini, and Pteropliini are paraphyletic. However, Shi et al. [10] believed that Acanthocinini, Agapanthiini, Dorcaschematini, Pteropliini, and Saperdini are monophyletic, whereas the monophyly of Lamiini is unstable. Our study is similar to the results of Souza et al. [17] and Ashman et al. [18] but differs from the data acquired by Shi et al. [10]. Overall, we prefer the results of Souza et al. [17] and Ashman et al. [18]. In this study, we collected the mitochondrial genomes of ten tribes: Acanthocinini, Agapanthiini, Apomecynini, Batocerini, Ceroplesini, Dorcaschematini, Lamiini, Mesosini, Pteropliini, and Saperdini. Dorcaschematini, Pteropliini, Agapanthiini, Ceroplesini, and Apomecynini have insufficient data to explore their monophyly. We believe that Acanthocinini is paraphyletic. In the Maximum Likelihood trees, *Acanthocinus griseus* (Acanthocinini) and Apomecynini clustered into one branch, and *Rondibilis* sp. (Acanthocinini) of our study clustered on the (Agapanthiini + Ceroplesini) branch. For Saperdini, based on the results of this study, we consider it to be monophyletic. Our study restored the monophyly of Saperdini, supporting Baltoceratid, Mesosini, Ceroplesini, and Lamiini as monophylic and Acanthocinini as paraphylic. The monophyly of Dorcaschematini, Pteropliini, Agapanthiini, and Apomecynini still needs to be researched.

## 5. Conclusions

We obtained 11 mitochondrial genomes of Lamiinae in this study. All gene sequences and compositions, except for *Batocera* sp., are relatively conserved, with no rearrangements or deletions. *Batocera* sp. contains a unique 1428-bp IGS with high A-T content and repetitive sequences that are similar to the A+T-rich region. Duplication/random loss of genes leads to the occurrence of a 269-bp IGS in *Me. myops*. In addition, we found that there is a consensus motif (TACTA) between trnS2 and nad1.

We supported the family rank of Oxypeltidae as well as the monophyly of the subfamilies Cerambycinae, Dorcasominae, Lamiinae, Lepturinae, Prioninae, Spondylidinae, Dorcasomidae, Vesperidae, and Disteniidae. Necydalinae (Necydalini) was downgraded to a tribe belonging to Lepturinae. Aseminae should be redefined to Spondylidinae. Our study restored the monophyly of Saperdini, supporting the monophyly of Batocerini, Ceroplesini, Lamiini, and Mesosini, and the paraphyly of Acanthocinini.

## Figures and Tables

**Table 1:** Statistical table of the number of subfamilies of the family Cerambycidae in three studies.

Author	Subfamily
Lawrence and Newton [6]	Anoplodermatinae, Apatophyseinae, Cerambycinae, Disteniinae, Oxypeltinae, Lepturinae, Lamiinae, Necydalinae, Parandrinae, Philinae, Prioninae, Spondylidinae, Vesperinae
Bouchard et al. [7]	Apatophyseinae, Cerambycinae, Dorcasominae, Lamiinae, Lepturinae, Necydalinae, Parandrinae, Prioninae, Spondylidinae
Svacha and Lawrence [8]	Cerambycinae, Dorcasominae, Lepturinae, Lamiinae, Necydalinae, Parandrinae, Prioninae, Spondylidinae

**Table 2:** Information on the 11 species from this study used in the phylogenetic analyses.

Species	Accession No.	Length (bp)	Tribe
*Acalolepta permutans*	OR149089	15,500	Lamiini
*Batocera* sp.	OR149086	16,843	Batocerini
*Eutetrapha metallescens*	OR149087	15,505	Saperdini
*Glenea pulchra*	OR149088	15,470	Saperdini
*Lamiomimus gottschei*	OR149090	16,421	Lamiini
*Macrochenus isabellinus*	OR149091	15,675	Lamiini
*Mesosa myops*	OR149092	18,499	Mesosini
*Oberea vittata*	OR149093	15,494	Saperdini
*Pharsalia subgemmata*	OR149094	16,553	Lamiini
*Rondibilis* sp.	OR149095	15,854	Acanthocinini
*Saperda subobliterata*	OR149096	15,499	Saperdini

## Data Availability

Data to support this study are available from the National Center for Biotechnology Information (https://www.ncbi.nlm.nih.gov) (accessed on 20 June 2023). GenBank numbers are OR98486-OR398496.

## Supplementary Materials

The following supporting information can be downloaded at: https://www.mdpi.com/article/10.3390/genes15010013/s1, Figures S1–S15: Figure S1: The maps of eleven newly sequenced mitochondrial genomes. Mitochondrial genomes located in the outer circle are encoded by the J-strand, and those located in the internal circle are encoded by the N-strand. The different kinds of tRNA are represented by amino acid abbreviations. GC content and GC skew are plotted as the deviation from the mean value of the entire sequence. Red boxes mean incomplete sequences; Figure S2: Eleven histograms of the relative synonymous codon usage (RSCU) in newly sequenced mitochondrial genomes. The X-axis shows synonymous codons, and the Y-axis shows RSCU values; Figure S3: The secondary structure of tRNAs in *Acalolepta permutans*; Figure S4: The secondary structure of tRNAs in *Batocera* sp.; Figure S5: The secondary structure of tRNAs in *Eutetrapha metallescens*; Figure S6: The secondary structure of tRNAs in *Glenea pulchra*; Figure S7: The secondary structure of tRNAs in *Lamiomimus gottschei*; Figure S8: The secondary structure of tRNAs in *Macrochenus isabellinus*; Figure S9: The secondary structure of tRNAs in *Mesosa myops*; Figure S10: The secondary structure of tRNAs in *Oberea vittate*; Figure S11: The secondary structure of tRNAs in *Pharsalia subgemmata*; Figure S12: The secondary structure of tRNAs in *Rondibilis* sp.; Figure S13: The secondary structure of tRNAs in *Saperda subobliterata*; Figure S14: Two types of Stem-loop structure of the long IGS in *Mesosa myops*; Figure S15: The proportions of the composition of the mitochondrial genomes. Green represents the coding region, yellow represents IGS, and pink represents the control region; Tables S1–S21: Table S1: Information on the 158 species used for phylogenetic analysis. Note: red words mean the newly sequenced mitochondrial genomes in this study; Table S2: Best partitioning scheme and best-fitting models selected for the 13 PCGs in the mitochondrial genome; Table S3: Location of features in the mitochondrial genome of 11 sequences; Table S4: Nucleotide composition and skewness of the whole mitochondrial genomes; Table S5: AT skew and GC skew of nucleotide sequences of 13 PCGs; Table S6: Comparative start and stop codons of newly sequenced genes. *Acalolepta permutans* (*A. permutans*), *Batocera* sp. (*B.* sp.), *Eutetrapha metallescens* (*E. metallescens*), *Glenea* *pulchra* (*G. pulchra*), *Lamiomimus gottschei* (*L. gottschei*), *Macrochenus isabellinus* (*Ma. isabellinus*), *Mesosa myops* (*Me. myops*), O*berea vittata* (*O. vittata*), *Pharsalia subgemmata* (*P. subgemmata*), *Rondibilis* sp. (*R.* sp.), *Saperda subobliterata* (*S. subobliterata*); Table S7: A+T content and length of the A+T-rich region of the 11 new sequences including the sites and length of poly-T and poly-A sequences in the A+T-rich region; Table S8: Repeats of A+T-rich region in *Acalolepta permutans*; Table S9: Repeats of A+T-rich region in *Batocera* sp.; Table S10: Repeats of A+T-rich region in *Eutetrapha metallescens*; Table S11: Repeats of A+T-rich region in *Glenea pulchra*; Table S12: Repeats of A+T-rich region in *Lamiomimus gottschei*; Table S13: Repeats of A+T-rich region in *Macrochenus isabellinus*; Table S14: Repeats of A+T-rich region in *Mesosa myops*; Table S15: Repeats of A+T-rich region in *Oberea vittate*; Table S16: Repeats of A+T-rich region in *Pharsalia subgemmata*; Table S17: Repeats of A+T-rich region in *Rondibilis* sp.; Table S18: Repeats of A+T-rich region in *Saperda subobliterata*; Table S19: Tandem repeats of long IGS of *Batocera* sp.; Table S20: Structure of long IGS of *Batocera* sp. and comparison with similar sequences; Table S21: Specific primers used to verify the long IGSs of *Batocera* sp. and *Mesosa myops*.
