# Supplementary material for: The Phylogenetic Relationship of Lamiinae (Coleoptera: Cerambycidae) Using Mitochondrial Genomes
Source: Genes (Basel). 2023 Dec 20;15(1):13. doi: 10.3390/genes15010013 (PMC10815127; doi:10.3390/genes15010013)
Supplement: Supplementary file 1 [file genes-15-00013-s001.zip › Table S1.pdf]

Table S1: Information on the 158 species used for phylogenetic analysis.

Accession No.	Subfamily	Spices	Length(bp)
**Cerambycidae**			
MN473105	Aseminae	*Arhopalus rusticus*	15,860
MW067122	Aseminae	*Arhopalus unicolor*	15,760
OM161964	Aseminae	*Cephalallus oberthueri*	15,763
KC751569	Cerambycinae	*Massicus raddei*	15,858
KM112084	Cerambycinae	*Xylotrechus grayii*	15,540
KX087339	Cerambycinae	*Pyrrhidium sanguineum*	16,203
KY796055	Cerambycinae	*Chlorophorus simillimus*	13,675
MK570750	Cerambycinae	*Xystrocera globosa*	15,707
MK863508	Cerambycinae	*Nortia carinicollis*	15,602
MN442320	Cerambycinae	*Pyrrhidium sanguineum*	15,748
MN473096	Cerambycinae	*Chlorophorus diadema*	15,398
MN473097	Cerambycinae	*Stenodryas* sp.	15,333
MN473098	Cerambycinae	*Trichoferus campestris*	15,737
MN473101	Cerambycinae	*Clytobius davidis*	15,571
MN473112	Cerambycinae	*Purpuricenus lituratus*	15,744
MT371041	Cerambycinae	*Aromia bungii*	15,760
MT525962	Cerambycinae	*Neoplocaederus obesus*	15,683
MT740323	Cerambycinae	*Epipedocera atra*	15,662
MT809475	Cerambycinae	*Anoplistes halodendri*	15,697
MW617355	Cerambycinae	*Aromia bungii*	15,652
MW858149	Cerambycinae	*Zoodes fulguratus*	15,885
MZ054170	Cerambycinae	*Jebusaea hammerschmidtii*	15,619
MZ707509	Cerambycinae	*Chlorophorus annularis*	15,487
OK189517	Cerambycinae	*Turanoclytus namaganensis*	15,565
OK393714	Cerambycinae	*Aromia bungii*	15,759
OL415177	Cerambycinae	*Aromia bungii*	15,760
OM069308	Cerambycinae	*Nadezhdiella cantori*	16,049
OM069309	Cerambycinae	*Allotraeus orientalis*	15,966
OM069310	Cerambycinae	*Xoanodera maculata*	15,767
OM645192	Cerambycinae	*Trachylophus sinensis*	15,746
OP096419	Cerambycinae	*Demonax pseudonotabilis*	15,527
KX035158	Disteniinae	*Disteniidae* sp.	15,598
MN473106	Disteniinae	*Distenia gracilis*	15,704
MN473107	Disteniinae	*Typodryas* sp.	15,647
MN473109	Disteniinae	*Clytomelegena kabakovi*	15,816
ON838309	Disteniinae	*Distenia punctulatoides*	15675
MN420488	Dorcasominae	*Tsivoka simplicicollis*	16,700
MN447435	Dorcasominae	*Dorcasomus pinheyi*	16,040

OR149089	Lamiinae	*Acalolepta permutans*	15,500
OR149086	Lamiinae	*Batocera* sp.	16,843
OR149087	Lamiinae	*Eutetrapha metallescens*	15,505
OR149088*	Lamiinae	*Glenea pulchra*	15,470
OR149090	Lamiinae	*Lamiomimus gottschei*	16,421
OR149091	Lamiinae	*Macrochenus isabellinus*	15,675
OR149092	Lamiinae	*Mesosa myops*	18,499
OR149093	Lamiinae	*Oberea vittata*	15,494
OR149094	Lamiinae	*Pharsalia subgemmata*	16,553
OR149095	Lamiinae	*Rondibilis* sp.	15,854
OR149096	Lamiinae	*Saperda subobliterata*	15,499
FJ424074	Lamiinae	*Psacothea hilaris*	15,856
JN986793	Lamiinae	*Batocera lineolata*	15,418
KJ809086	Lamiinae	*Monochamus alternatus*	15,874
KT726932	Lamiinae	*Anoplophora chinensis*	15,805
KX184801	Lamiinae	*Apriona swainsoni*	15,412
KY773692	Lamiinae	*Agapanthia daurica*	14,282
KY796054	Lamiinae	*Olenecamptus subobliteratus*	13,854
MF521888	Lamiinae	*Batocera lineolata*	15,420
MK423971	Lamiinae	*Aristobia reticulator*	15,838
MK863507	Lamiinae	*Blepephaeus succinctor*	15,554
MK863509	Lamiinae	*Oberea yaoshana*	15,529
MK863510	Lamiinae	*Pterolophia* sp.	16,063
MK863511	Lamiinae	*Thermistis croceocincta*	15,503
MN044086	Lamiinae	*Glenea cantor*	15,514
MN248534	Lamiinae	*Anoplophora horsfieldi*	15,796
MN356095	Lamiinae	*Annamanum lunulatum*	15,610
MN473099	Lamiinae	*Acanthocinus griseus*	15,600
MN473100	Lamiinae	*Niphona lateraliplagiata*	15,902
MN473114	Lamiinae	*Agapanthia daurica*	17,153
MN473116	Lamiinae	*Epiglenea comes*	15,213
MN473117	Lamiinae	*Glenea licenti*	15,435
MN473119	Lamiinae	*Menesia sulphurata*	15,551
MN610562	Lamiinae	*Apomecyna saltator*	14,949
MN709785	Lamiinae	*Oberea diversipes*	15,499
MN882586	Lamiinae	*Anoplophora chinensis*	15,871
MT547196	Lamiinae	*Monochamus alternatus*	15,880
MT740324	Lamiinae	*Olenecamptus bilobus*	15,262
MW067123	Lamiinae	*Agelasta perplexa*	15,552
MW067124	Lamiinae	*Monochamus sparsutus*	16,029
MW364565	Lamiinae	*Anoplophora horsfieldi*	15,837

MW617354	Lamiinae	*Agapanthia amurensis*	15,512
MW617356	Lamiinae	*Moechotypa diphysis*	15,493
MW629558	Lamiinae	*Batocera lineolata*	16,158
MW858148	Lamiinae	*Paraglenea fortunei*	15,496
MW858150	Lamiinae	*Aulaconotus atronotatus*	14,491
MW858151	Lamiinae	*Apriona germarii*	14,858
MW858152	Lamiinae	*Monochamus alternatus*	14,189
MZ073344	Lamiinae	*Batocera lineolata*	15,420
MZ955033	Lamiinae	*Saperda tetrastigma*	15,563
NC013070	Lamiinae	*Psacothea hilaris*	15,866
OK030905	Lamiinae	*Morimospasma tuberculatum*	15,485
OM161963	Lamiinae	*Batocera rubus*	16,158
ON641973	Lamiinae	*Pseudoechthistatus hei*	16,103
OP006455	Lamiinae	*Pseudoechthistatus chiangshunani*	16,419
OP169419	Lamiinae	*Monochamus saltuarius*	15,896
OP169420	Lamiinae	*Monochamus sartor urussovii*	15,912
OP613265	Lamiinae	*Sthenias yunnanus*	15,633
KX087246	Lepturinae	*Brachyta interrogationis*	18,165
KX087264	Lepturinae	*Cortodera humeralis*	15,928
KX087348	Lepturinae	*Stenurella nigra*	16,504
KY773687	Lepturinae	*Anastrangalia sequensi*	16,269
MN420469	Lepturinae	*Leptura annularis*	16,530
MN473080	Lepturinae	*Grammoptera ruficornis*	16,458
MN473082	Lepturinae	*Stenocorus meridianus*	16,227
MN473095	Lepturinae	*Peithona prionoides*	13,636
MN473103	Lepturinae	*Rhagium fortecostatum*	16,274
MN473111	Lepturinae	*Teledapalpus zolotichini*	16,651
MN473113	Lepturinae	*Sachalinobia koltzei*	15,809
MN473110	Necydalinae	*Ulochaetes vacca*	15,593
MN420466	Oxypeltimae	*Oxypeltus quadrispinosus*	17,001
KY773686	Prioninae	*Aegosoma sinicum*	15,658
MF521835	Prioninae	*Callipogon relictus*	15,742
MG460483	Prioninae	*Dorysthenes paradoxus*	15,922
MK614532	Prioninae	*Howea angulata*	15,626
MK614533	Prioninae	*Olethrius laevipennis*	15,690
MK614534	Prioninae	*Utra nitida*	14,976
MK614535	Prioninae	*Phaolus metallicus*	15,997
MK614536	Prioninae	*Cnemoplites australis*	15,675
MK614537	Prioninae	*Bifidoprionus rufus*	15,590
MK614538	Prioninae	*Aesa media*	15,714
MK614540	Prioninae	*Rhipidocerus australasiae*	15,721

MK614541	Prioninae	*Brephilydia jejuna*	15,659
MK614542	Prioninae	*Hermerius prionoides*	13,696
MK614543	Prioninae	*Cacodacnus planicollis*	15,671
MK614544	Prioninae	*Geoffmonteithia queenslanda*	15,628
MK614545	Prioninae	*Enneaphyllus aeneipennis*	16,505
MK614546	Prioninae	*Eboraphyllus middletoni*	15,776
MK614547	Prioninae	*Eurynassa australis*	15,612
MK614548	Prioninae	*Toxeutes arcuatus*	15,859
MK614549	Prioninae	*Pseudoplites inexpectatus*	15,651
MK614550	Prioninae	*Agrianome spinicollis*	15,633
MK614551	Prioninae	*Analophus parallelus*	15,722
MK614552	Prioninae	*Xixuthrus* sp.	15,523
MK614553	Prioninae	*Teispes insularis*	15,632
MK614554	Prioninae	*Archetypus frenchi*	16,156
MK614555	Prioninae	*Sceleocantha* sp.	15,804
MK614556	Prioninae	*Cnemoplites edulis*	13,161
MK614559	Prioninae	*Toxeutes macleayi*	13,579
MN420471	Prioninae	*Aegolipton marginale*	16,759
MN420482	Prioninae	*Nepiodes costipennis multicarinatus*	15,935
MN473090	Prioninae	*Tragosoma depsarium*	15,712
MN473091	Prioninae	*Sarmydus* sp.	15,720
MN473102	Prioninae	*Phlyctenosis* sp.	15,000
MN473115	Prioninae	*Aegosoma pallidum*	15,668
MN594765	Prioninae	*Megopis sinica*	15,689
MN607219	Prioninae	*Paulhutchinsonia pilosicollis*	15,846
MN829437	Prioninae	*Dorysthenes granulosus*	15,858
OM161965	Prioninae	*Priotyrannus closteroides*	15,854
MN473088	Spondylidinae	*Saphanus piceus*	15,832
MN420476	Spondylinae	*Spondylis buprestoides*	16,070
MN473093	Vesperinae	*Vesperus sanzi*	16,125
MT826860	Spondylinae	*Spondylis buprestoides*	15,837
**Vesperidae**			
KT781589		*Spiniphilus spinicornis*	15,707
MN473092		*Mantitheus pekinensis*	15,656
MN473120		*Philus antennatus*	15,833
MZ747394		*Philus pallescens*	15,750
**Cucujidae**			
MK614518		*Platisus angusticollis*	15,921
MK614524		*Palaestes abruptus*	15,534
NC051939		*Cucujus kempi*	15,492

Note: red text indicates the newly sequenced mitochondrial genomes in this study.
