# Supplementary material for: The Phylogenetic Relationship of Lamiinae (Coleoptera: Cerambycidae) Using Mitochondrial Genomes
Source: Genes (Basel). 2023 Dec 20;15(1):13. doi: 10.3390/genes15010013 (PMC10815127; doi:10.3390/genes15010013)
Supplement: Supplementary file 1 [file genes-15-00013-s001.zip › Table S10.pdf]

Table S10: Repeats of A+T-rich region in *Eutetrapha metallescens*.

Indices	Period Size	Copy Number	Consensus Size	Percent Matches
319-500	2	102.0	2	65
327-503	13	14.2	13	67
344-503	17	9.1	17	64
319-515	42	4.8	42	71
321-533	46	4.5	46	71
311-520	29	6.7	32	71
