# Supplementary material for: The Phylogenetic Relationship of Lamiinae (Coleoptera: Cerambycidae) Using Mitochondrial Genomes
Source: Genes (Basel). 2023 Dec 20;15(1):13. doi: 10.3390/genes15010013 (PMC10815127; doi:10.3390/genes15010013)
Supplement: Supplementary file 1 [file genes-15-00013-s001.zip › Table S11.pdf]

Table S11: Repeats of A+T-rich region in *Glenea pulchra*.

Indices	Period Size	Copy Number	Consensus Size	Percent Matches
274-456	8	21.7	9	67
371-456	2	46.0	2	71
274-456	17	10.4	19	67
354-449	15	6.7	15	73
373-473	30	3.3	32	77
368-459	15	6.0	15	70
416-512	40	2.3	42	81
