# Supplementary material for: The Phylogenetic Relationship of Lamiinae (Coleoptera: Cerambycidae) Using Mitochondrial Genomes
Source: Genes (Basel). 2023 Dec 20;15(1):13. doi: 10.3390/genes15010013 (PMC10815127; doi:10.3390/genes15010013)
Supplement: Supplementary file 1 [file genes-15-00013-s001.zip › Table S12.pdf]

Table S12: Repeats of A+T-rich region in *Lamiomimus gottschei*.

Indices	Period Size	Copy Number	Consensus Size	Percent Matches
164--300	41	3.1	46	61
118--1052	86	10.9	86	96
336--558	41	5.1	46	60
594--730	41	3.1	46	61
766--988	41	5.1	46	61
812--1028	41	5.1	40	61
1272--1319	19	2.6	18	83
1287--1359	11	7.4	10	69
1295--1391	17	5.7	17	70
1279--1385	47	2.3	47	79
