# Supplementary material for: The Phylogenetic Relationship of Lamiinae (Coleoptera: Cerambycidae) Using Mitochondrial Genomes
Source: Genes (Basel). 2023 Dec 20;15(1):13. doi: 10.3390/genes15010013 (PMC10815127; doi:10.3390/genes15010013)
Supplement: Supplementary file 1 [file genes-15-00013-s001.zip › Table S13.pdf]

Table S13: Repeats of A+T-rich region in *Macrochenus isabellinus*.

Indices	Period Size	Copy Number	Consensus Size	Percent Matches
3-111	57	1.9	58	98
536-620	27	3.1	29	74
