# Supplementary material for: The Phylogenetic Relationship of Lamiinae (Coleoptera: Cerambycidae) Using Mitochondrial Genomes
Source: Genes (Basel). 2023 Dec 20;15(1):13. doi: 10.3390/genes15010013 (PMC10815127; doi:10.3390/genes15010013)
Supplement: Supplementary file 1 [file genes-15-00013-s001.zip › Table S14.pdf]

Table S14: Repeats of A+T-rich region in *Mesosa myops*.

Indices	Period Size	Copy Number	Consensus Size	Percent Matches
242-275	15	2.2	16	89
637-670	15	2.2	16	89
1-2768	395	7.0	395	100
1032-1065	15	2.2	16	89
1427-1460	15	2.2	16	89
1822-1855	15	2.2	16	89
2217-2250	15	2.2	16	89
2612-2645	15	2.2	16	89
2985-3023	18	2.1	19	86
3409-3464	10	5.6	10	97
3451-3499	21	2.3	21	100
3449-3489	12	3.7	12	75
