# Supplementary material for: The Phylogenetic Relationship of Lamiinae (Coleoptera: Cerambycidae) Using Mitochondrial Genomes
Source: Genes (Basel). 2023 Dec 20;15(1):13. doi: 10.3390/genes15010013 (PMC10815127; doi:10.3390/genes15010013)
Supplement: Supplementary file 1 [file genes-15-00013-s001.zip › Table S15.pdf]

Table S15: Repeats of A+T-rich region in *Oberea vittata*.

Indices	Period Size	Copy Number	Consensus Size	Percent Matches
276-384	43	2.6	41	83
295-483	15	13.1	14	66
394-459	23	2.8	23	84
391-461	2	38.5	2	70
357-485	16	8.1	15	69
605-643	19	2.1	19	85
