# Supplementary material for: The Phylogenetic Relationship of Lamiinae (Coleoptera: Cerambycidae) Using Mitochondrial Genomes
Source: Genes (Basel). 2023 Dec 20;15(1):13. doi: 10.3390/genes15010013 (PMC10815127; doi:10.3390/genes15010013)
Supplement: Supplementary file 1 [file genes-15-00013-s001.zip › Table S16.pdf]

Table S16: Repeats of A+T-rich region in *Pharsalia subgemmata*.

Indices	Period Size	Copy Number	Consensus Size	Percent Matches
460-1092	71	8.9	71	100
1375-1515	2	79.0	2	66
1372-1433	15	4.5	14	73
1372-1509	26	5.5	24	83
1396-1481	23	3.7	22	74
1375-1510	25	5.3	26	83
1372-1514	48	3.0	47	79
1419-1524	44	2.4	44	79
1576-1619	19	2.2	20	84
