# Supplementary material for: The Phylogenetic Relationship of Lamiinae (Coleoptera: Cerambycidae) Using Mitochondrial Genomes
Source: Genes (Basel). 2023 Dec 20;15(1):13. doi: 10.3390/genes15010013 (PMC10815127; doi:10.3390/genes15010013)
Supplement: Supplementary file 1 [file genes-15-00013-s001.zip › Table S17.pdf]

Table S17: Repeats of A+T-rich region in *Rondibilis* sp..

| Indices | Period Size | Copy Number | Consensus Size | Percent Matches |
|---|---|---|---|---|
| 62-91 | 15 | 2.0 | 15 | 100 |
| 214-345 | 41 | 3.2 | 40 | 73 |
| 192-357 | 82 | 2.0 | 82 | 98 |
| 266-338 | 32 | 2.3 | 32 | 79 |
| 455-532 | 32 | 2.5 | 31 | 81 |
| 489-829 | 19 | 19.9 | 18 | 73 |
| 508-831 | 44 | 7.3 | 44 | 91 |
| 480-835 | 132 | 2.7 | 132 | 92 |
| 681-838 | 21 | 7.4 | 21 | 71 |
