# Supplementary material for: The Phylogenetic Relationship of Lamiinae (Coleoptera: Cerambycidae) Using Mitochondrial Genomes
Source: Genes (Basel). 2023 Dec 20;15(1):13. doi: 10.3390/genes15010013 (PMC10815127; doi:10.3390/genes15010013)
Supplement: Supplementary file 1 [file genes-15-00013-s001.zip › Table S18.pdf]

Table S18: Repeats of A+T-rich region in *Saperda subobliterata*.

| Indices | Period Size | Copy Number | Consensus Size | Percent Matches |
|---|---|---|---|---|
| 317-498 | 2 | 99.0 | 2 | 66 |
| 309-465 | 23 | 7.2 | 23 | 66 |
| 315-515 | 25 | 7.8 | 25 | 68 |
| 338-515 | 15 | 11.5 | 15 | 70 |
| 342-501 | 17 | 9.1 | 17 | 69 |
