# Supplementary material for: The Phylogenetic Relationship of Lamiinae (Coleoptera: Cerambycidae) Using Mitochondrial Genomes
Source: Genes (Basel). 2023 Dec 20;15(1):13. doi: 10.3390/genes15010013 (PMC10815127; doi:10.3390/genes15010013)
Supplement: Supplementary file 1 [file genes-15-00013-s001.zip › Table S19.pdf]

Table S19: Tandem repeats of long IGS of *Batocera* sp.

Indices	Period Size	Copy Number	Consensus Size	Percent Matches
14--1428	80	17.7	80	97
14--1428	319	4.4	319	97
