# Supplementary material for: The Phylogenetic Relationship of Lamiinae (Coleoptera: Cerambycidae) Using Mitochondrial Genomes
Source: Genes (Basel). 2023 Dec 20;15(1):13. doi: 10.3390/genes15010013 (PMC10815127; doi:10.3390/genes15010013)
Supplement: Supplementary file 1 [file genes-15-00013-s001.zip › Table S2.pdf]

Table S2: Best partitioning scheme and best-fitting models selected of mitochondrial 13 PCGs

Nucleotide Aequence Alignments		
**Subset**	**Best Model**	**Partition names**
1	GTR+I+G	*cytb*_codon1, *atp6*_codon1
2	TVM+I+G	*cytb*_codon2, *atp6*_codon2, *nad3*_codon2
3	TVM+G	*cox2*_codon3, *atp6*_codon3
4	GTR+I+G	*atp8*_codon2, *nad3*_codon1, *atp8*_codon1, *nad6*_codon1
5	GTR+G	*atp8*_codon3, *nad6*_codon3
6	GTR+I+G	*cox1*_codon1, *cox3*_codon1
7	TVM+I+G	*cox2*_codon2, *cox3*_codon2, *cox1*_codon2
8	GTR+I+G	*nad3*_codon3, *cox1*_codon3
9	GTR+I+G	*cox2*_codon1
10	GTR+I+G	*cox3*_codon3, *cytb*_codon3
11	GTR+I+G	*nad4*_codon1, *nad5*_codon1, *nad4l*_codon1, *nad1*_codon1
12	GTR+I+G	*nad4l*_codon2, *nad1*_codon2, *nad4*_codon2, *nad5*_codon2
13	TVM+I+G	*nad4*_codon3, *nad1*_codon3, *nad4l*_codon3
14	GTR+I+G	*nad2*_codon1
15	GTR+I+G	*nad6*_codon2, *nad2*_codon2
16	TRN+G	*nad2*_codon3
17	TRN+I+G	*nad5*_codon3
