# Supplementary material for: The Phylogenetic Relationship of Lamiinae (Coleoptera: Cerambycidae) Using Mitochondrial Genomes
Source: Genes (Basel). 2023 Dec 20;15(1):13. doi: 10.3390/genes15010013 (PMC10815127; doi:10.3390/genes15010013)
Supplement: Supplementary file 1 [file genes-15-00013-s001.zip › Table S20.pdf]

Table S20: Structure of long IGS of *Batocera* sp. and comparison with similar sequence.

Specie	Length	A+T	Position	Structure
Pyrocoelia rufa	1,724bp	69.5%	*nad2*, W	12 tandem repeats, 1 repeat of the beginning.
Evania appendigaster	534 bp	96.8%	K, D	3 types of tandem repeats (Each type has 7 copies, 12 copies, and 8 copies, respectively);<br>1 initial sequence;<br>1 terminal sequence.
Metopodontus blanchardi	4,051bp	65.4%	I , Q	17 tandem repeat sequences (15 copies are complete, 2 units are incomplete).
Batocera sp.	1,428bp	72%	W, C	18 tandem repeat sequences(17 copies are complete, 1 unit is incomplete).
