# Supplementary material for: The Phylogenetic Relationship of Lamiinae (Coleoptera: Cerambycidae) Using Mitochondrial Genomes
Source: Genes (Basel). 2023 Dec 20;15(1):13. doi: 10.3390/genes15010013 (PMC10815127; doi:10.3390/genes15010013)
Supplement: Supplementary file 1 [file genes-15-00013-s001.zip › Table S21.pdf]

Table S21: Specific primers used to verify the long IGSs of *Batocera* sp. and *Mesosa myops*.

Name	Sequence
YNWS-8-J-1252	GTAAACTATCAACCTTCAAA
YNWS-8-N-3425	CCTGCTAAAACTGGTAATG
YNWS-8-J-1091	TCTACGAATCACCTTTACAG
YNWS-8-N-2814	GCCGAGTTTAAGCAGTAA
JLWJD9-J-NAD	ATTCTACACCAATCACAAA
JLWJD9-N-NAD	AAGCACCAAATAAGAAGT
