# Supplementary material for: The Phylogenetic Relationship of Lamiinae (Coleoptera: Cerambycidae) Using Mitochondrial Genomes
Source: Genes (Basel). 2023 Dec 20;15(1):13. doi: 10.3390/genes15010013 (PMC10815127; doi:10.3390/genes15010013)
Supplement: Supplementary file 1 [file genes-15-00013-s001.zip › Table S6.pdf]

Table S6: Comparative start and stop codons of newly sequenced genes. *Acalolepta permutans* (*A. permutans*), *Batocera* sp. (*B.* sp.), *Eutetrapha metallescens* (*E. metallescens*), *Glenea pulchra* (*G. pulchra*), *Lamiomimus gottschei* (*L. gottschei*), *Macrochenus isabellinus* (*Ma. isabellinus*), *Mesosa myops* (*Me. myops*), *Oberea vittata* (*O. vittata*), *Pharsalia subgemmata* (*P. subgemmata*), *Rondibilis* sp (*R.* sp.), *Saperda subobliterata* (*S. subobliterata*).

	atp6	***atp8***	***cox1***	***cox2***	***cox3***	***cytb***	***nad1***	***nad2***	***nad3***	***nad4***	***nad4l***	***nad5***	***nad6***
A. permutans	ATG/TAA	ATT/TAA	AAC/T	ATT/T	ATG/TAA	ATG/TAG	TTG/TAG	ATT/TAA	ATA/TAG	ATG/TA	ATG/TAA	ATT/TA	ATT/TAA
B. sp.	ATG/TAA	ATT/TAA	AAA/T	ATC/T	ATG/TA	ATG/TAG	TTG/TAG	ATT/TAA	ATC/TAG	ATG/TA	ATG/TAA	ATT/TA	ATT/TAA
E. metallescens	ATG/TAA	ATT/TAA	AAC/T	ATT/T	ATG/TAA	ATG/TAG	TTG/TAG	ATC/TAA	ATT/TAG	ATG/TA	ATG/TAA	ATA/TA	ATT/TAA
G. pulchra	ATG/TAA	ATT/TAA	AAC/T	ATT/T	ATG/TAA	ATG/TAG	TTG/TAG	ATT/TAA	ATT/TAG	ATG/TA	ATG/TAA	ATG/T	ATT/TAA
L. gottschei	ATG/TAA	ATT/TAA	AAC/TAA	ATT/T	ATG/TAA	ATG/TAG	TTG/TAG	ATC/TAA	ATT/TAG	ATG/TA	ATG/TAA	ATT/TAA	ATT/TAA
Ma. isabellinus	ATG/TAA	ATT/TAG	AAC/T	ATC/T	ATG/TAA	ATG/TAA	TTG/TAG	ATT/TAA	ATT/TAG	ATG/TA	ATG/TAA	ATT/TA	ATT/TAA
Me. myops	ATG/TAA	ATT/TAA	ATT/T	ATT/T	ATG/TAA	ATG/TAG	TTG/TAG	ATC/TAA	ATT/TAG	ATG/TAA	ATG/TAA	ATT/TA	ATT/TAA
O. vittata	ATG/TAA	ATT/TAA	AAC/T	ATT/T	ATG/TAA	ATG/TAG	TTG/TAG	ATT/TAA	ATT/TAG	ATG/TA	ATG/TAA	ATT/TA	ATA/TAA
P. subgemmata	ATG/TAA	ATT/TAG	AAC/T	ATT/T	ATG/TA	ATG/TAG	TTG/TAG	ATT/TAA	ATA/TAG	ATG/TA	ATG/TAA	ATT/TA	ATT/TAA
R. sp.	ATG/TAA	ATC/TAA	AAC/T	ATT/T	ATG/TAG	ATG/TAG	TTG/TAG	ATT/TAA	ATT/TAG	ATG/TAA	ATG/TAA	ATT/TA	ATT/TAA
S. subobliterata	ATG/TAA	ATT/TAA	AAC/TAA	ATT/T	ATG/TAA	ATG/TAG	TTG/TAG	ATC/TAA	ATC/TAG	ATG/TA	ATG/TAA	ATT/TA	ATC/TAA
