# Supplementary material for: The Phylogenetic Relationship of Lamiinae (Coleoptera: Cerambycidae) Using Mitochondrial Genomes
Source: Genes (Basel). 2023 Dec 20;15(1):13. doi: 10.3390/genes15010013 (PMC10815127; doi:10.3390/genes15010013)
Supplement: Supplementary file 1 [file genes-15-00013-s001.zip › Table S7.pdf]

Table S7: A+T content and length of the A+T-rich region of the 11 new sequences including the sites and length of poly-T and poly-A sequences in the A+T-rich region.

Species	A+T content	Length	T-stretch sites	T-stretch length(bp)
Acalolepta permutans	87%	878	288-298	11
			788-820	33
Batocera sp.	85%	736	153-166	14
Eutetrapha metallescens	86%	869	283-293	11
			722-733	12
Glenea pulchra	87%	813	238-248	11
			678-686	9
Lamiomimus gottschei	78%	1763	1,242-1,254	13
			1,472-1480	9
			1,693-1703	11
Macrochenus isabellinus	82%	1011	431-444	14
		**A-stretch**	931-946	16
Mesosa myops	80%	3,560	2,937-2,948	12
			3,459-3,467	9
			3,480-3,488	9
			3,529-3,537	9
Oberea vittata	87%	829	248-259	12
Pharsalia subgemmata	86%	1922	1,336-1,349,	14
			1,630-1,638	9
			1,855-1,864	10
			1,878-1,887	10
			1,899-1,907	9
		**A-stretch**	25-33	9
			1,780-1,788	9
Rondibilis sp.	90%	1,236	374-388	15
			1,116-1124	
Saperda subobliterata	86%	866	282-290	9
			720-729	
