# Supplementary material for: The Phylogenetic Relationship of Lamiinae (Coleoptera: Cerambycidae) Using Mitochondrial Genomes
Source: Genes (Basel). 2023 Dec 20;15(1):13. doi: 10.3390/genes15010013 (PMC10815127; doi:10.3390/genes15010013)
Supplement: Supplementary file 1 [file genes-15-00013-s001.zip › Table S8.pdf]

Table S8: Repeats of A+T-rich region in *Acalolepta permutans*.

Indices	Period Size	Copy Number	Consensus Size	Percent Matches
101-141	19	2.2	19	90
318-408	44	2.0	44	81
444-533	2	48.0	2	67
482-538	23	2.6	21	76
788-820	1	33.0	1	100
