# Supplementary material for: The Phylogenetic Relationship of Lamiinae (Coleoptera: Cerambycidae) Using Mitochondrial Genomes
Source: Genes (Basel). 2023 Dec 20;15(1):13. doi: 10.3390/genes15010013 (PMC10815127; doi:10.3390/genes15010013)
Supplement: Supplementary file 1 [file genes-15-00013-s001.zip › Table S9.pdf]

Table S9: Repeats of A+T-rich region in *Batocera* sp..

Indices	Period Size	Copy Number	Consensus Size	Percent Matches
205-249	19	2.4	19	92
326-396	25	2.8	26	81
323-398	27	2.9	27	79
