# Supplementary figures and images for: The Phylogenetic Relationship of Lamiinae (Coleoptera: Cerambycidae) Using Mitochondrial Genomes
Source: Genes (Basel). 2023 Dec 20;15(1):13. doi: 10.3390/genes15010013 (PMC10815127; doi:10.3390/genes15010013)

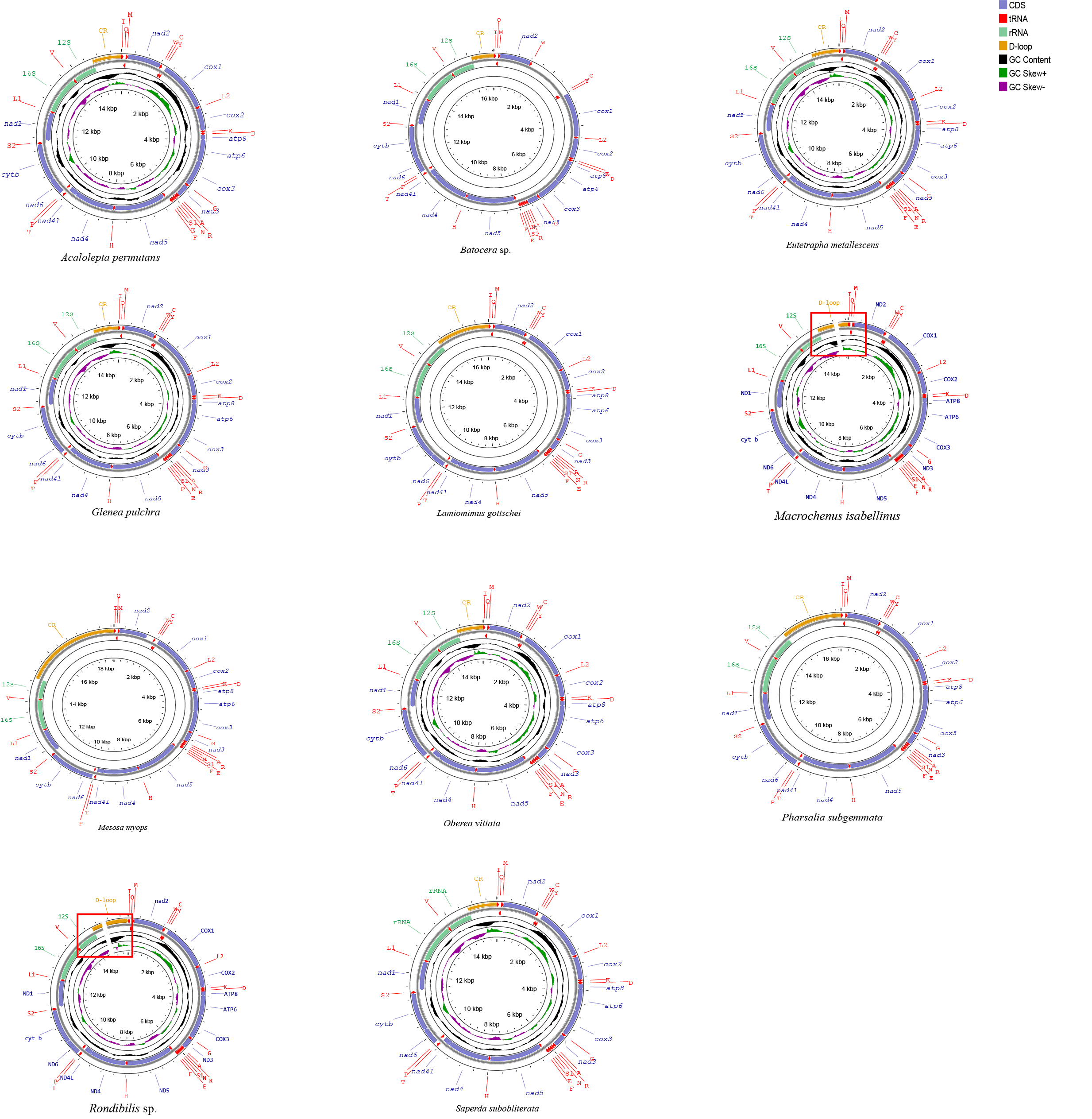

Supplement: Supplementary file 1 [file genes-15-00013-s001.zip › Figure S1.png]

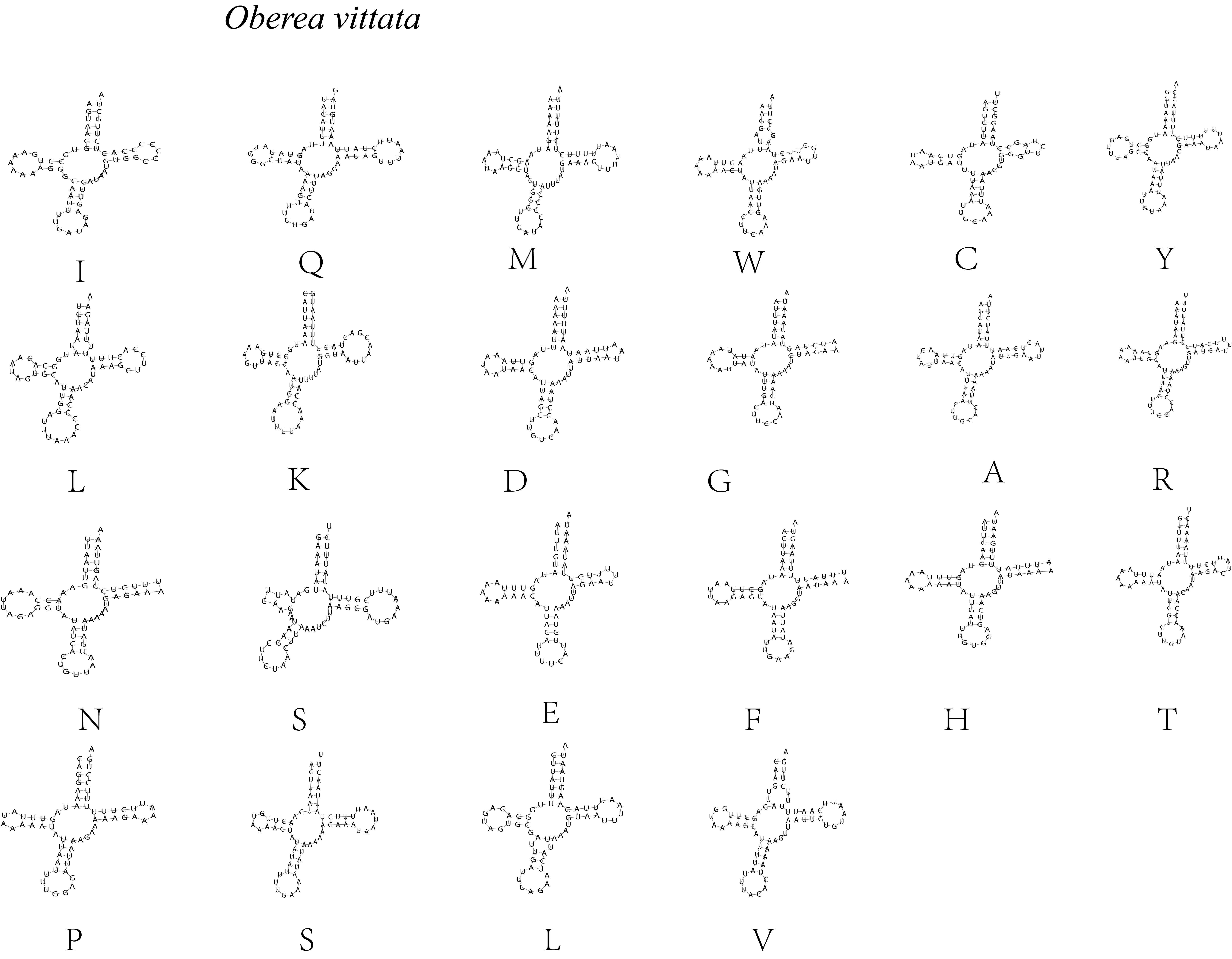

Supplement: Supplementary file 1 [file genes-15-00013-s001.zip › Figure S10.png]

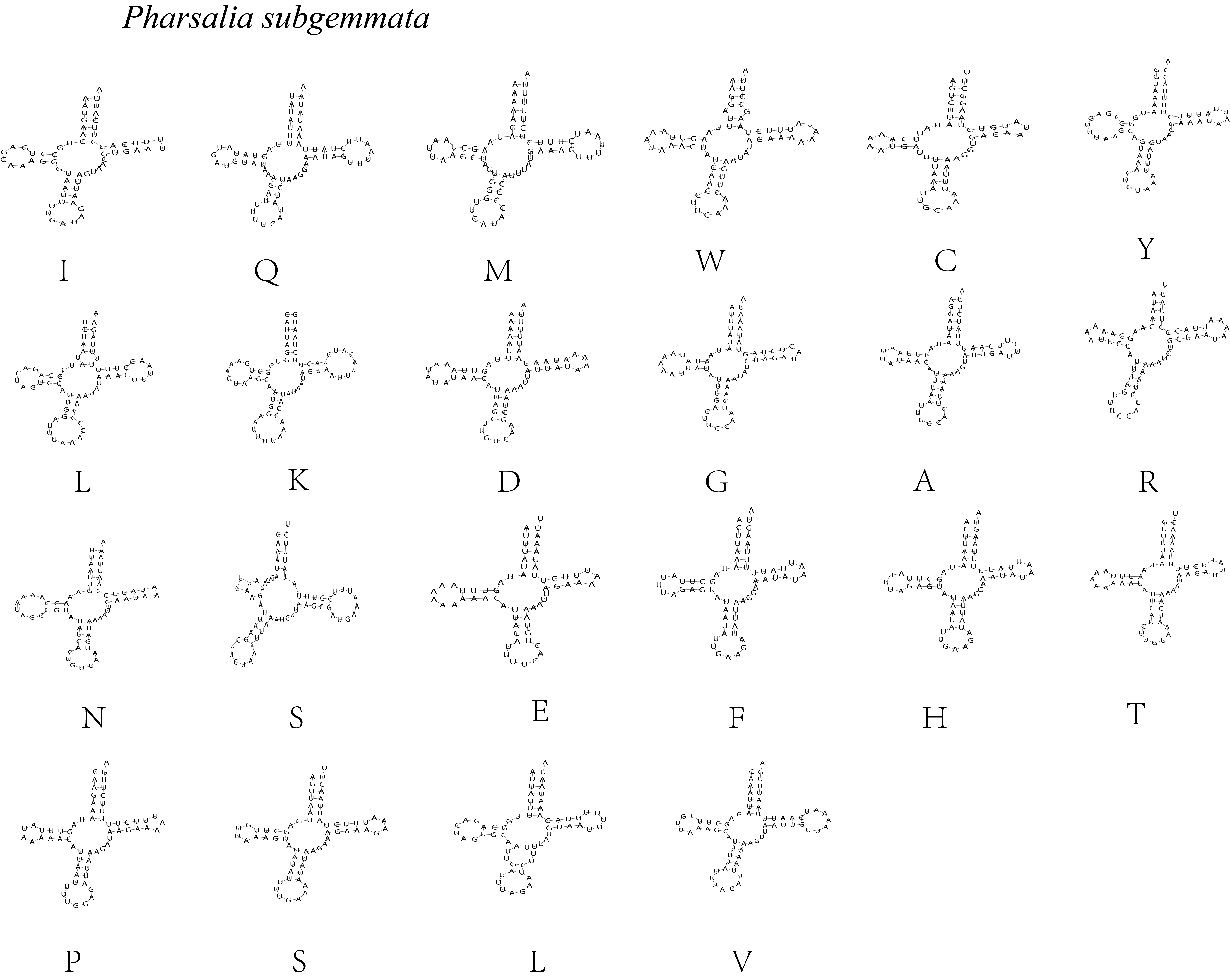

Supplement: Supplementary file 1 [file genes-15-00013-s001.zip › Figure S11.png]

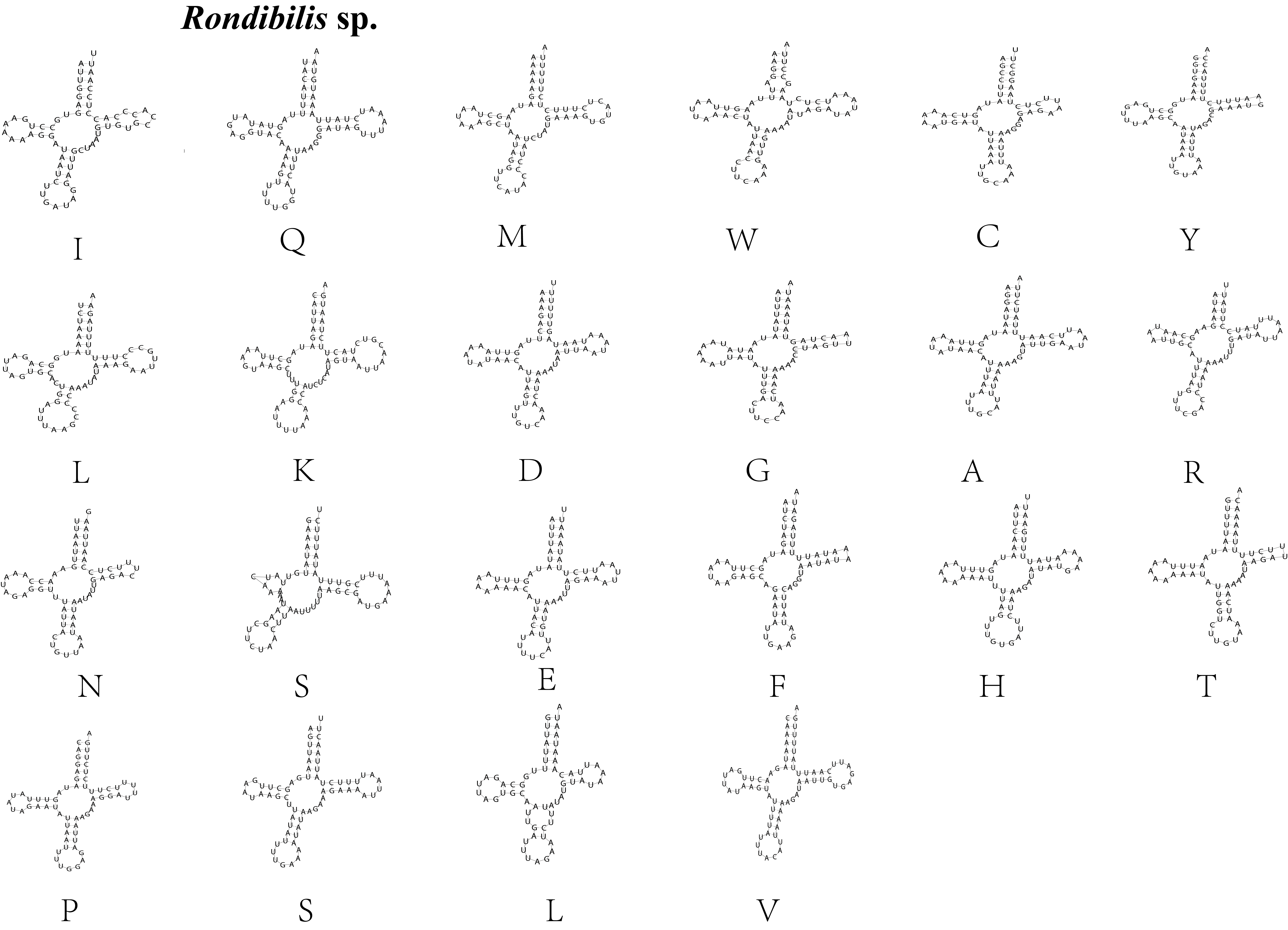

Supplement: Supplementary file 1 [file genes-15-00013-s001.zip › Figure S12.png]

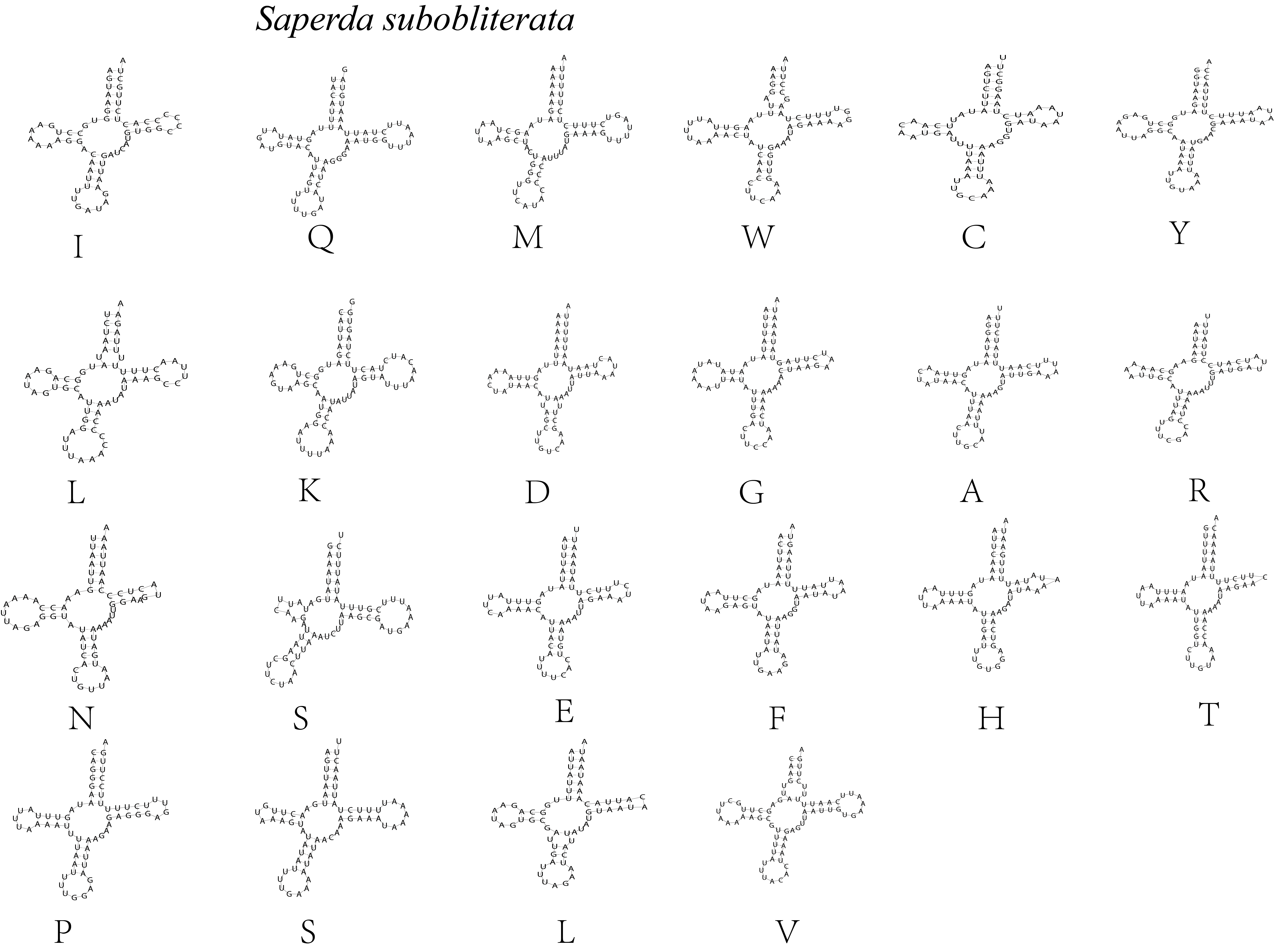

Supplement: Supplementary file 1 [file genes-15-00013-s001.zip › Figure S13.png]

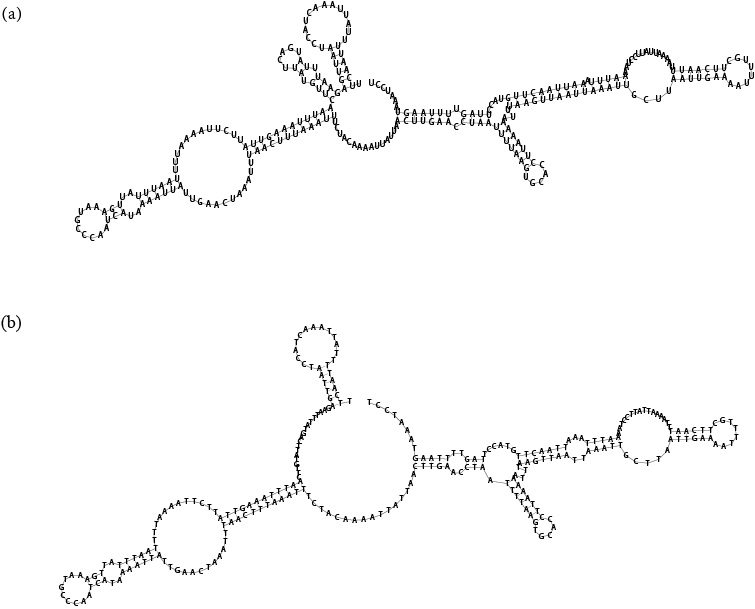

Supplement: Supplementary file 1 [file genes-15-00013-s001.zip › Figure S14.png]

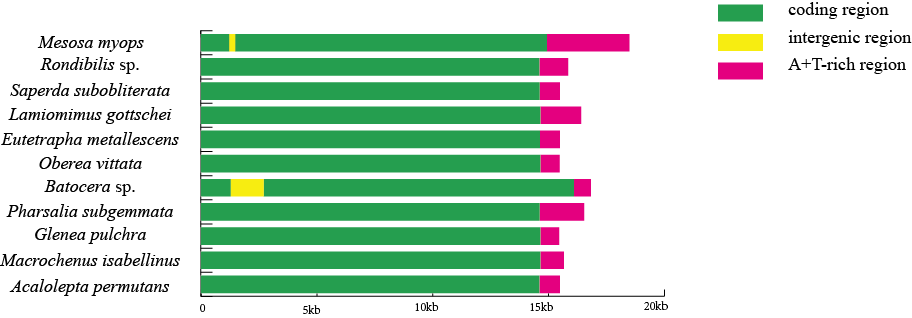

Supplement: Supplementary file 1 [file genes-15-00013-s001.zip › Figure S15.png]

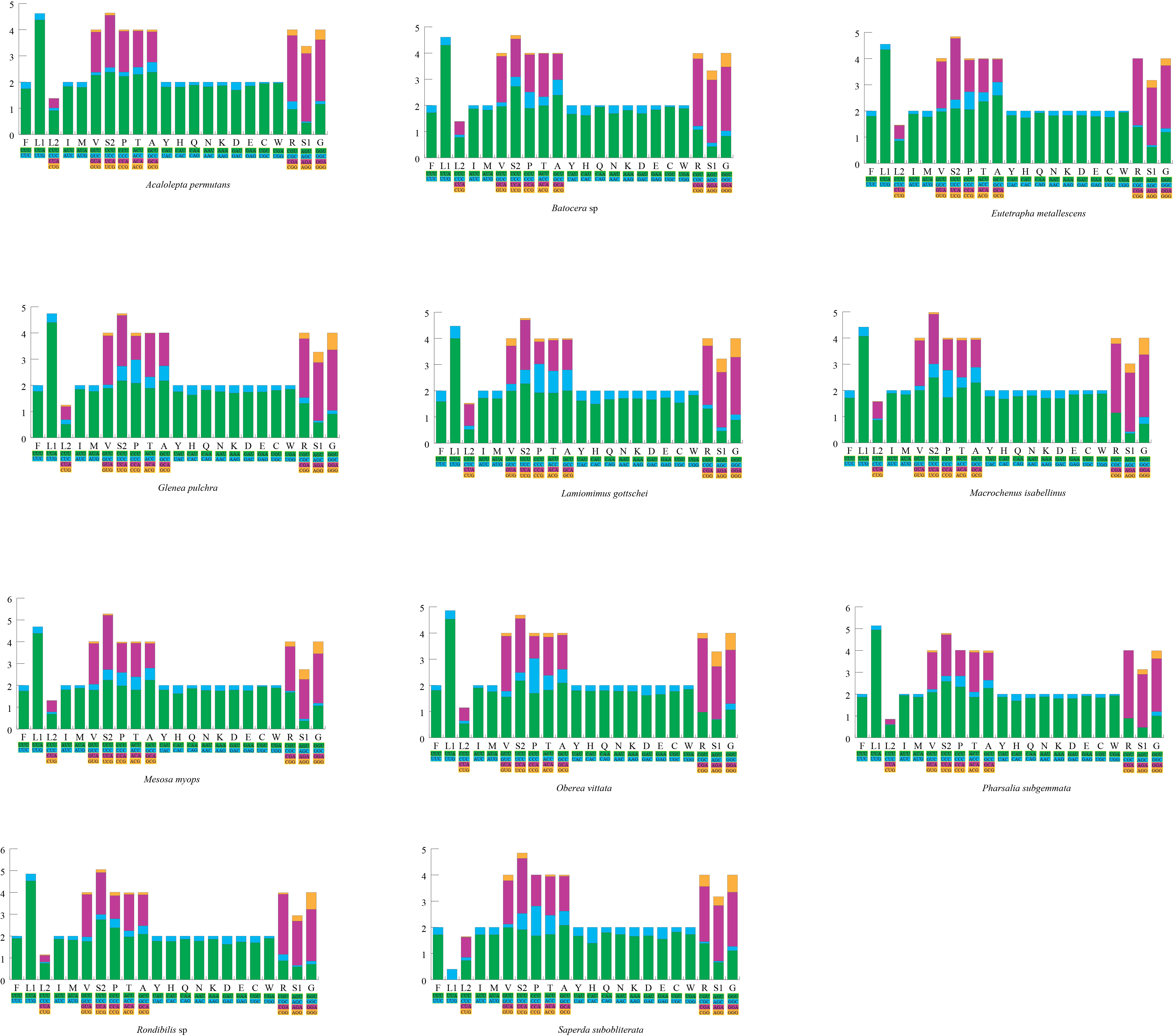

Supplement: Supplementary file 1 [file genes-15-00013-s001.zip › Figure S2.png]

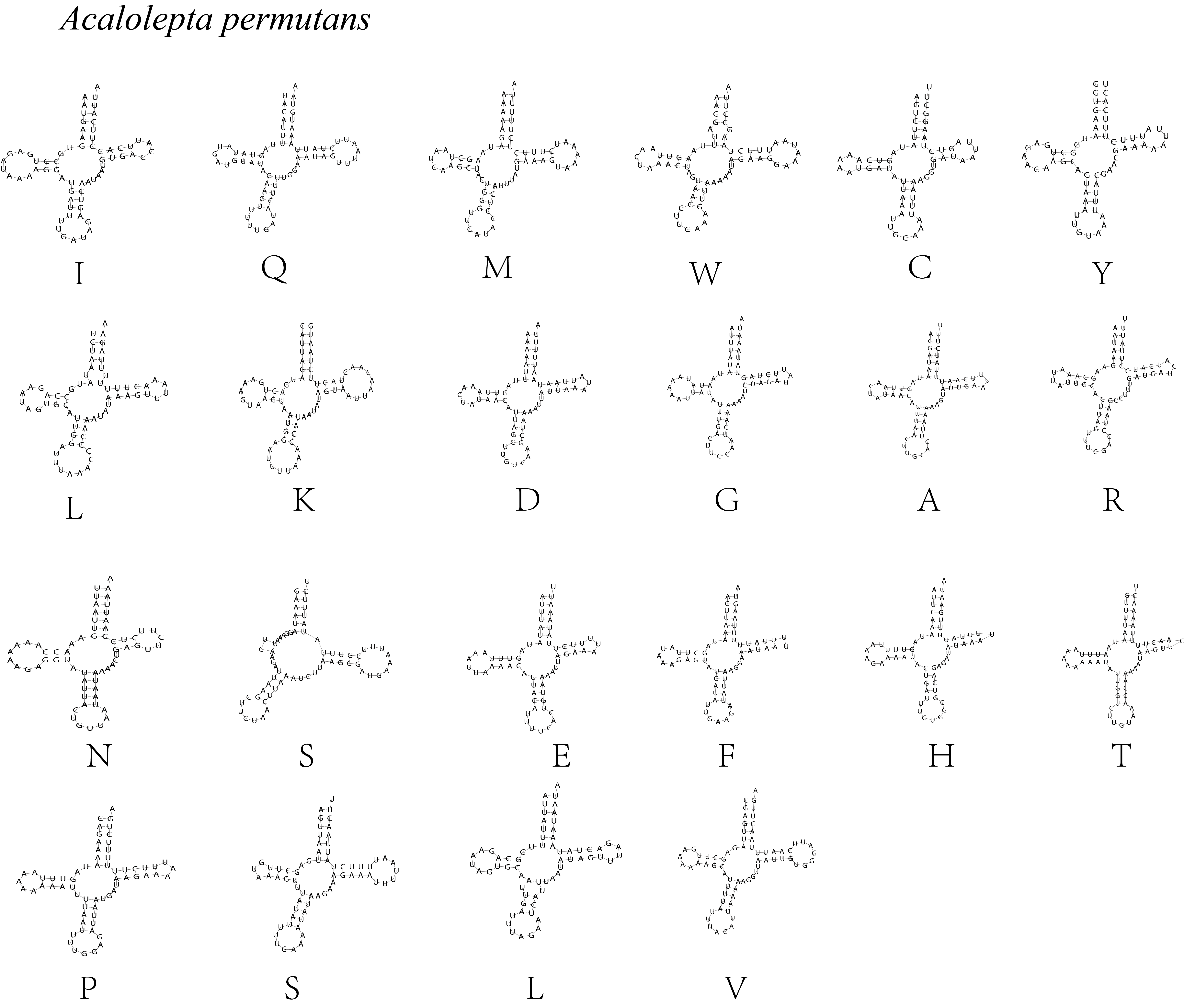

Supplement: Supplementary file 1 [file genes-15-00013-s001.zip › Figure S3.png]

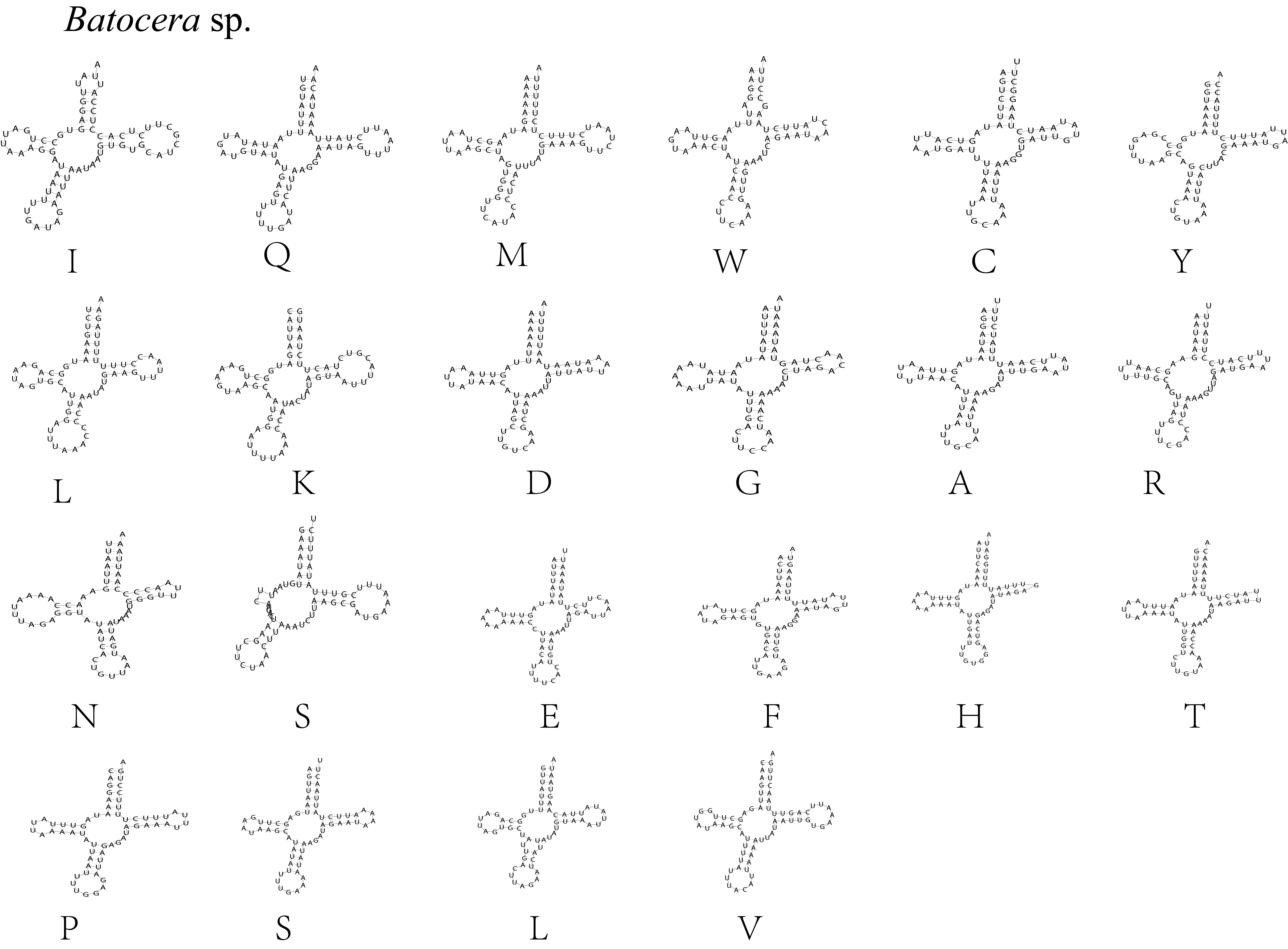

Supplement: Supplementary file 1 [file genes-15-00013-s001.zip › Figure S4.png]

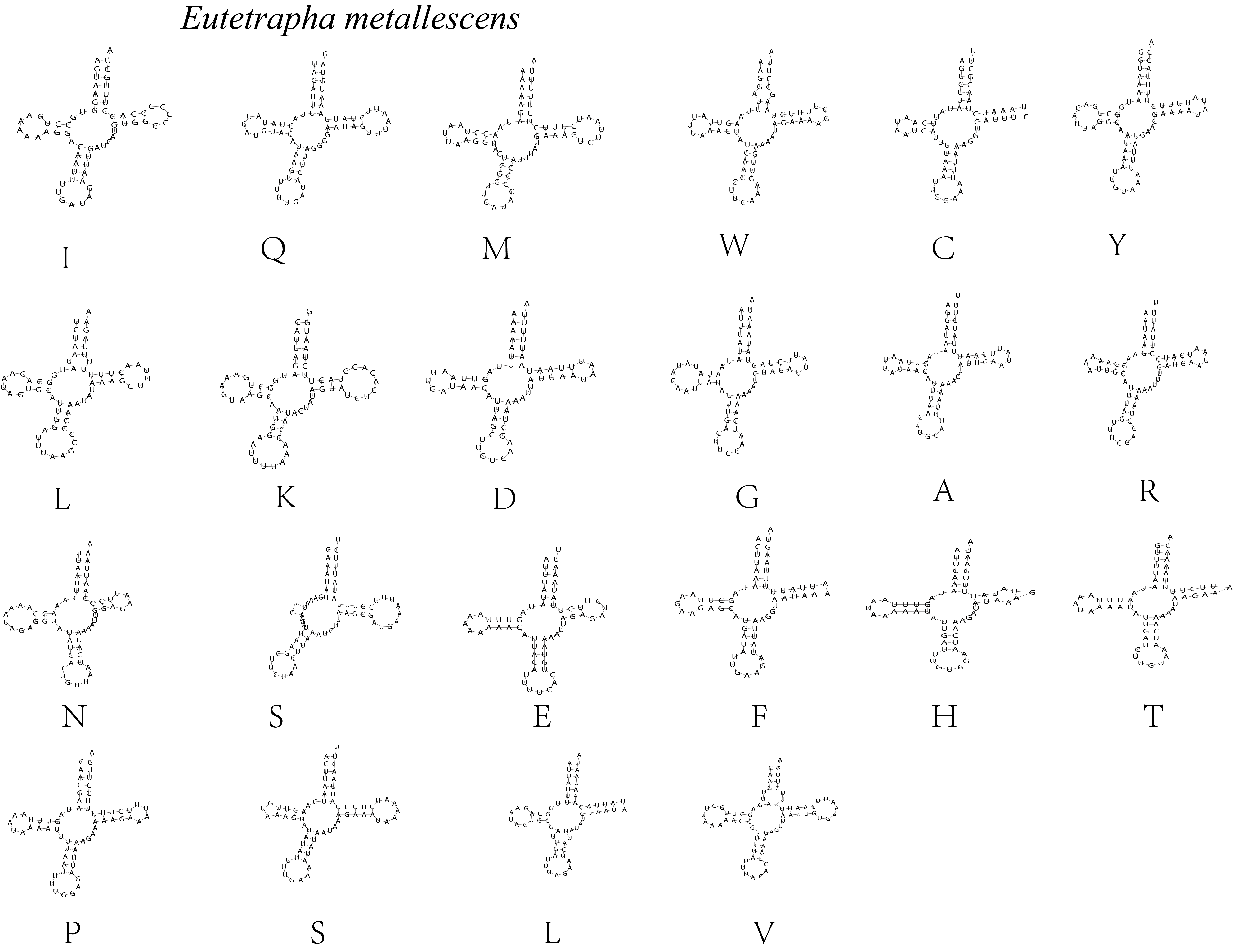

Supplement: Supplementary file 1 [file genes-15-00013-s001.zip › Figure S5.png]

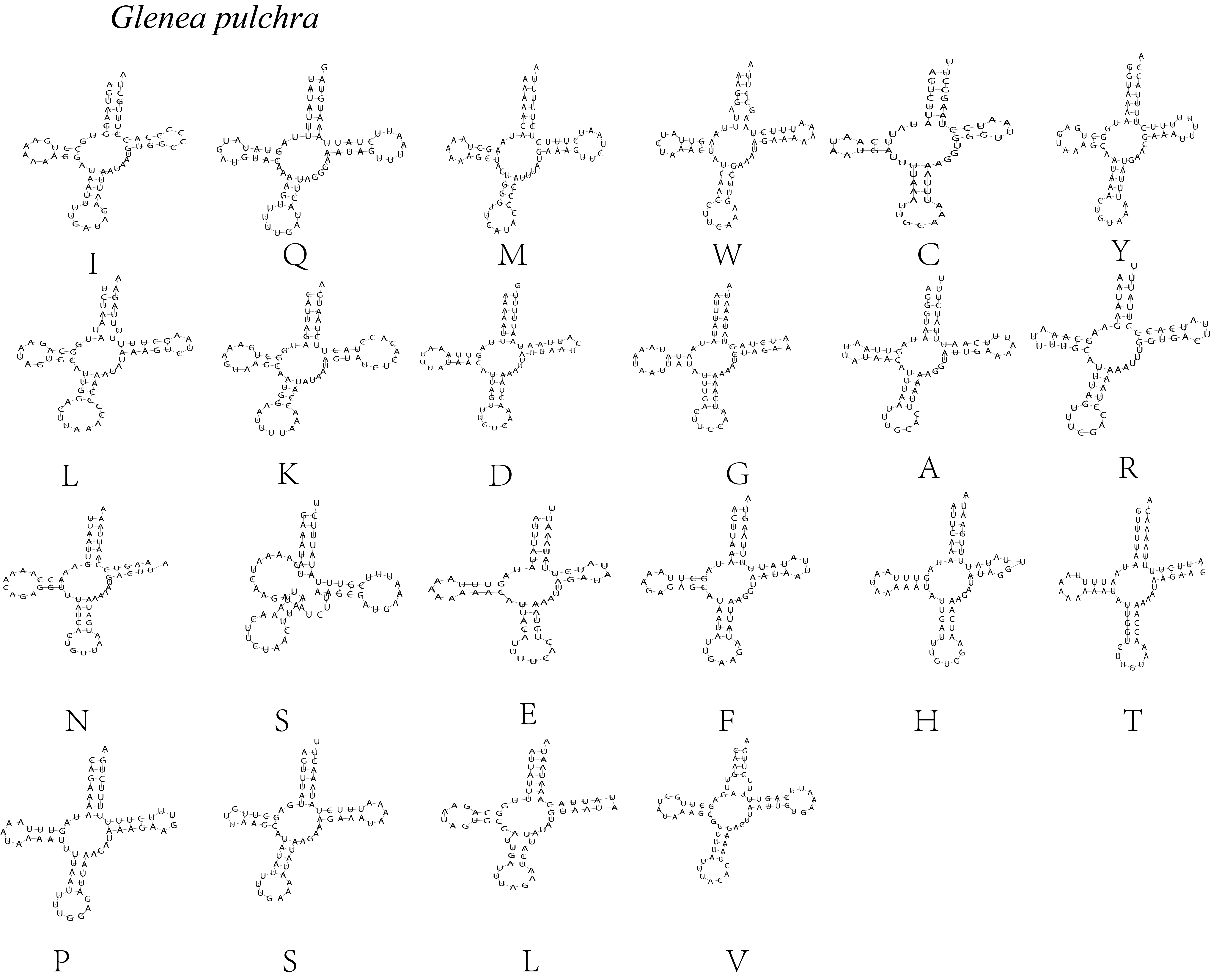

Supplement: Supplementary file 1 [file genes-15-00013-s001.zip › Figure S6.png]

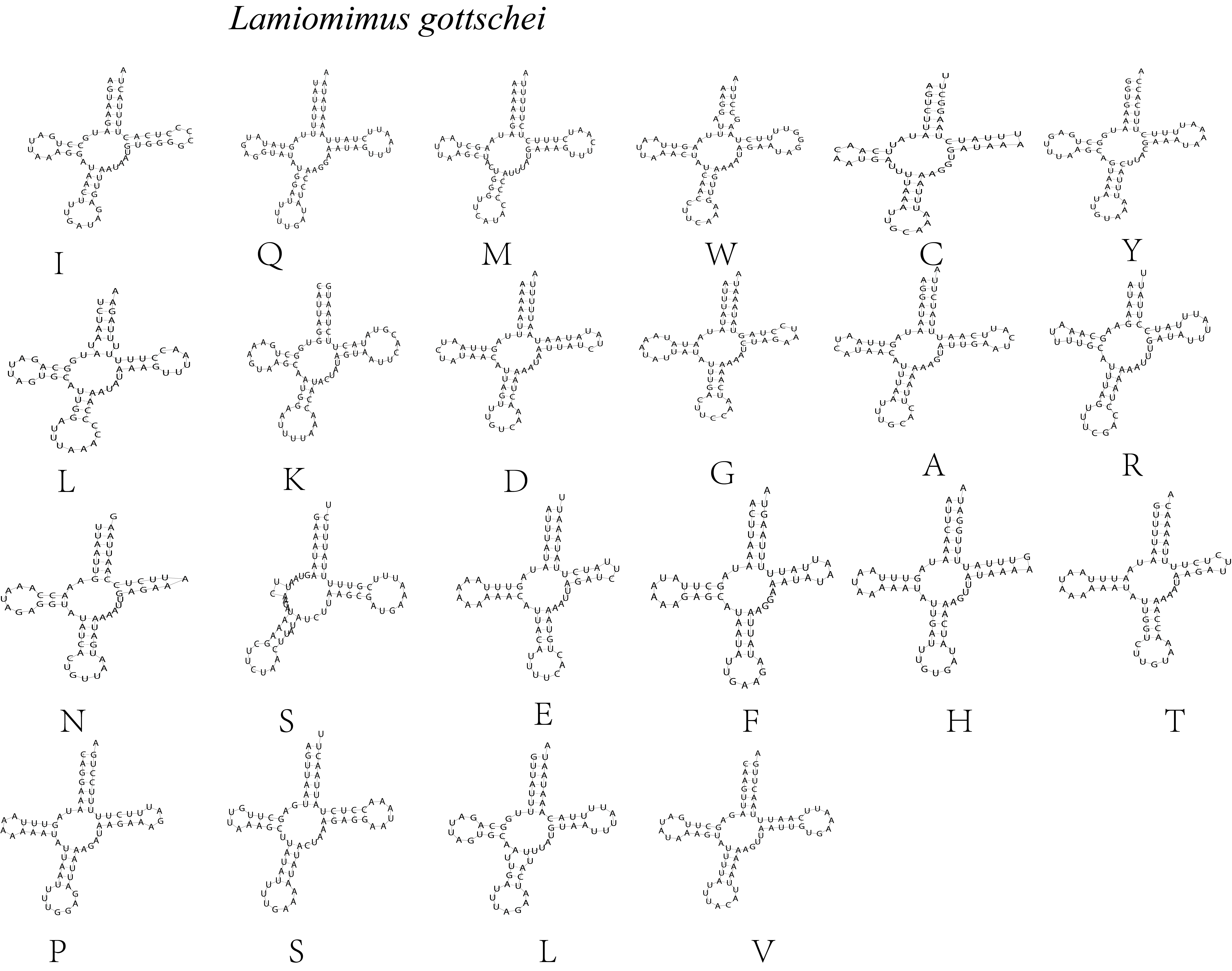

Supplement: Supplementary file 1 [file genes-15-00013-s001.zip › Figure S7.png]

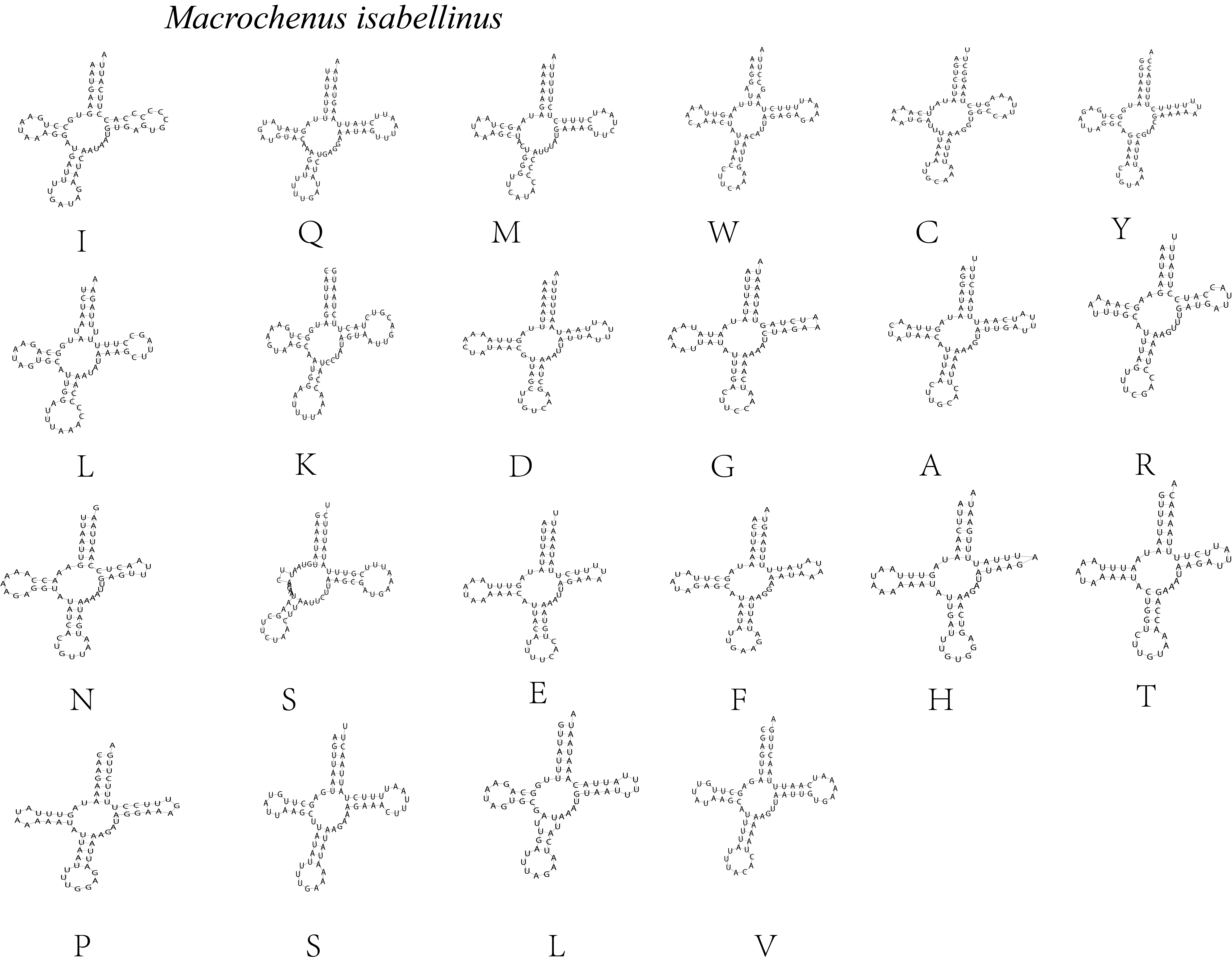

Supplement: Supplementary file 1 [file genes-15-00013-s001.zip › Figure S8.png]

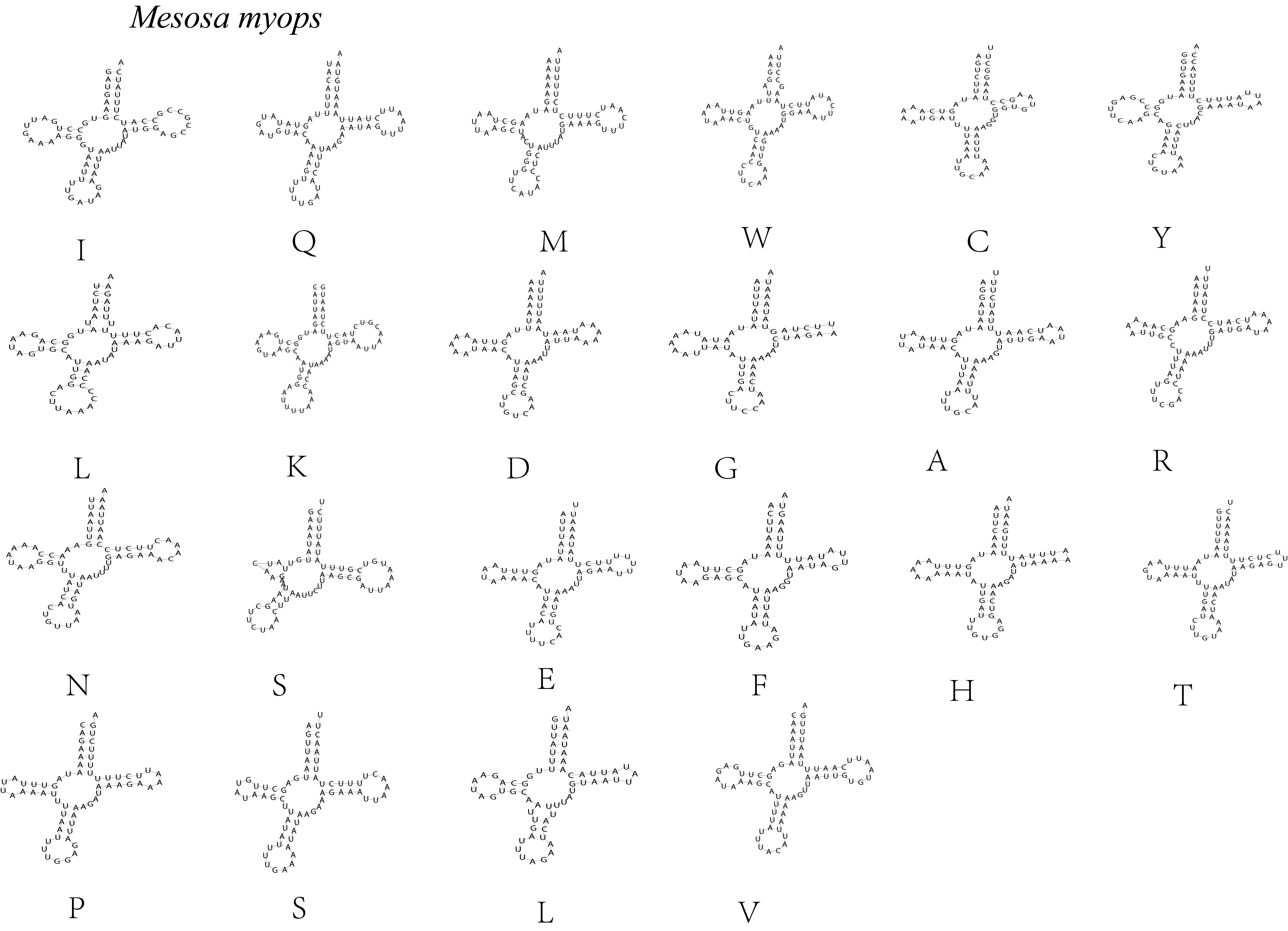

Supplement: Supplementary file 1 [file genes-15-00013-s001.zip › Figure S9.png]
